# Characterizing long COVID in an international cohort: 7 months of symptoms and their impact

**DOI:** 10.1016/j.eclinm.2021.101019

**Published:** 2021-07-15

**Authors:** Hannah E. Davis, Gina S. Assaf, Lisa McCorkell, Hannah Wei, Ryan J. Low, Yochai Re'em, Signe Redfield, Jared P. Austin, Athena Akrami

**Affiliations:** a Patient-Led Research Collaborative; b Sainsbury Wellcome Centre, University College London, London, United Kingdom; c Department of Psychiatry, NewYork-Presbyterian Hospital / Weill Cornell Medicine, NYC, United States; d Oregon Health and Science University, Portland, OR, United States

**Keywords:** Long COVID, Post-acute Sequelae of COVID-19, PASC, Patient-Led research, COVID-19, COVID recovery, COVID-19 symptoms, Post-COVID-19 Syndrome, Post Acute COVID, Long Hauler

## Abstract

**Background:**

A significant number of patients with COVID-19 experience prolonged symptoms, known as Long COVID. Few systematic studies have investigated this population, particularly in outpatient settings. Hence, relatively little is known about symptom makeup and severity, expected clinical course, impact on daily functioning, and return to baseline health.

**Methods:**

We conducted an online survey of people with suspected and confirmed COVID-19, distributed via COVID-19 support groups (e.g. Body Politic, Long COVID Support Group, Long Haul COVID Fighters) and social media (e.g. Twitter, Facebook). Data were collected from September 6, 2020 to November 25, 2020. We analyzed responses from 3762 participants with confirmed (diagnostic/antibody positive; 1020) or suspected (diagnostic/antibody negative or untested; 2742) COVID-19, from 56 countries, with illness lasting over 28 days and onset prior to June 2020. We estimated the prevalence of 203 symptoms in 10 organ systems and traced 66 symptoms over seven months. We measured the impact on life, work, and return to baseline health.

**Findings:**

For the majority of respondents (>91%), the time to recovery exceeded 35 weeks. During their illness, participants experienced an average of 55.9+/- 25.5 (mean+/-STD) symptoms, across an average of 9.1 organ systems. The most frequent symptoms after month 6 were fatigue, post-exertional malaise, and cognitive dysfunction. Symptoms varied in their prevalence over time, and we identified three symptom clusters, each with a characteristic temporal profile. 85.9% of participants (95% CI, 84.8% to 87.0%) experienced relapses, primarily triggered by exercise, physical or mental activity, and stress. 86.7% (85.6% to 92.5%) of unrecovered respondents were experiencing fatigue at the time of survey, compared to 44.7% (38.5% to 50.5%) of recovered respondents. 1700 respondents (45.2%) required a reduced work schedule compared to pre-illness, and an additional 839 (22.3%) were not working at the time of survey due to illness. Cognitive dysfunction or memory issues were common across all age groups (~88%). Except for loss of smell and taste, the prevalence and trajectory of all symptoms were similar between groups with confirmed and suspected COVID-19.

**Interpretation:**

Patients with Long COVID report prolonged, multisystem involvement and significant disability. By seven months, many patients have not yet recovered (mainly from systemic and neurological/cognitive symptoms), have not returned to previous levels of work, and continue to experience significant symptom burden.

**Funding:**

All authors contributed to this work in a voluntary capacity. The cost of survey hosting (on Qualtrics) and publication fee was covered by AA's research grant (Wellcome Trust/Gatsby Charity via Sainsbury Wellcome center, UCL).

**Research in context**

**Evidence before this study**

Several studies have confirmed the presence of persistent symptoms following acute infection with COVID-19. Most recently, a large study conducted within the United States Veteran Affairs Health Care System found that patients with acute COVID-19 experienced higher rates of morbidity and mortality over the ensuing six months following diagnosis compared to uninfected individuals. This study, and many preceding it, utilized administrative databases and ICD-10 codes to identify and categorize these sequelae, which may inadvertently simplify the complexity of the Long COVID patient experience and miss details that can only be captured through direct patient assessment.

**Added value of this study**

This patient-directed study examines the largest collection of symptoms identified in the Long COVID population to date, is the first to quantify individual symptom trajectories over an extended period of time, and demonstrates the large impact symptoms have on patients’ ability to work and perform daily tasks. The comprehensive assay of symptoms spans 10 organ systems (neuropsychiatric, systemic, reproductive, cardiovascular, musculoskeletal, immunological, head-ear-eye-nose-throat, pulmonary, gastrointestinal, and dermatologic). Cluster analysis reveals that symptoms share common modes of variation in their prevalence over time, and that symptoms with similar time courses are distributed across multiple organ systems. A combination of the neurological/cognitive and systemic symptoms are shown to persist the longest.

**Implications of all the available evidence**

Given the millions of cases of COVID-19 worldwide and current research showing one in seven COVID-19 patients still symptomatic at 12 weeks, the number of Long COVID patients is likely substantial. The results of this study suggest Long COVID is composed of heterogeneous sequelae that often affect multiple organ systems, with significant impacts on morbidity, mortality, and quality of life. Given the heterogeneity of Long COVID, multidisciplinary research will be necessary to understand the pathophysiology of the disease and develop effective treatments. This research also highlights the importance of slowing the spread of COVID-19 through validated public health measures and vaccinations, and highlights the necessity of a robust safety net including sick leave, family leave, disability benefits, and workplace protections and flexibilities.

Alt-text: Unlabelled box

## 1 Introduction

Public discourse on COVID-19 has largely centered around those with severe or fatal illness [1]. However, recent studies show that a growing number of patients with initially mild COVID-19 will experience prolonged symptoms [2,3], the profile and timeline of which remains uncertain [2,[4], [5], [6], [7], [8], [9]]. Early in the course of the pandemic, patients identified this trend, referring to themselves as “Long-Haulers” and the prolonged illness as “Long COVID”[10]. There exist few systematic studies investigating this population, and relatively little is known about the range of symptom makeup and severity, expected clinical course, impact on daily functioning, and expected return to baseline health [11].

While as of yet there is no agreed upon case definition of Long COVID [8,12], we define the illness as a collection of symptoms that develop during or following a confirmed or suspected case of COVID-19, and which continue for more than 28 days. This is a similar definition to the Centers for Disease Control and Prevention's (CDC) “Post-COVID conditions”[13].

In this paper, we report results from an online survey investigating the symptoms of Long COVID in patients with illness onset between December 2019 and May 2020, allowing analysis of symptoms over 7 months’ duration. The aim of this study is to better describe the patient experience and recovery process in those with confirmed or suspected COVID-19 illness, with a specific emphasis on the Long COVID experience. The unique approach of this study utilizes patient-driven research [14] in order to establish a foundation of evidence for medical investigation, improvement of care [15,16], and advocacy for the Long COVID population. In this study, we investigate the patient's lived experience, emphasizing symptom course and severity over time with an in-depth look into neurological and neuropsychiatric symptoms, recovery, and return to baseline, including work impact. Other topics investigated in the survey will be included in future reports.

## 2 Methods

### 2.1 Study design

The survey was created by a team of patients with COVID-19 who are members of the Body Politic online COVID-19 support group and formed the Patient-Led Research Collaborative. The group conducted its first survey in April 2020 and issued a subsequent report in May 2020 [7]. The second survey was created to investigate details of recovery, testing results, the impact on mental health, and a more comprehensive set of symptoms with a greater emphasis on neurological symptoms. During the curation of survey questions, we worked closely with other patients to compile the list of symptoms, design research questions on how the Long COVID condition may affect daily life of the patients, and optimize the questionnaire design to reduce survey fatigue.

The survey was launched on September 6, 2020. Data were collected using Qualtrics (www.qualtrics.com), an online survey platform. All respondents gave digital informed consent prior to participating. Survey responses contained no personally identifiable information, and email addresses collected for survey distribution were encrypted as anonymized participant IDs. The study was approved by the University College London (UCL) Research Ethics Committee [16,159.002] (London, UK), and Oregon Health and Science University Institutional Review Board (IRB) (Portland, OR, USA), with UCL serving as the primary site. The Weill Cornell Medical College IRB determined non-engagement.

The survey consisted of 257 questions and required a median time of 69.3 min to complete. To account for Long COVID symptoms that limit sustained focus and attention, respondents were encouraged to take breaks while completing the survey. Progress was saved for up to 30 days to allow respondents to return to the survey at a later time. Questions that mentioned technical terms included a description in plain language.

The survey was created in English and translated into eight additional languages: Spanish, French, Portuguese, Italian, Dutch, Russian, Bahasa Indonesian, and Arabic. Links to the survey were disseminated via email, social media, and the online patient support groups listed in Appendix C.4. Data included in the analysis were collected from September 6 to November 25, 2020.

### 2.2 Study population (Inclusion criteria)

The survey "Information Sheet" (accessible here: patientresearchcovid19.com/survey2) stated: "You are being invited to participate in this research study because you have had a COVID-19, or suspected COVID-19 infection (still suffering or suffered symptoms) for longer than 1 week and you are 18 years of age or older.” All respondents consented to these criteria. To characterize Long COVID symptoms over an extended period, analysis was limited to respondents with illness lasting longer than 28 days and symptom onset between December 2019 and May 2020.

Methods used to distribute the survey did not allow us to determine the number of people who viewed the invitation. The proxy response rate was measured as the ratio of those who completed to those who started the survey. A total of 7285 responses were downloaded from the Qualtrics server on November 25, 2020. The following responses were removed from the dataset: incomplete (those not reaching the end of the survey, *n* = 2367), no illness onset date (*n* = 2), onset date before December 2019 (*n* = 26), 0 days of symptoms (*n* = 1), duplicate participants (*n* = 150), symptoms for 28 days or less (*n* = 401), and illness onset after May 2020 (*n* = 576). This resulted in complete data from 3762 respondents.

One of the questions in the survey asked about the annual income of the participant's household. Options were provided based on five income quintiles in USA (USD), Canada (CAD), United Kingdom (GBP), and Europe (EURO). 3084 (82.0%) respondents reported their income at the time of the survey, from which their socioeconomic status was estimated (Appendix C.1, Figure S1).

In addition to positively tested subjects [*n* = 1020, either diagnostic (RT-PCR/antigen) or antibody, Table 1], we included participants with absent (*n* = 1819) or negative test results (*n* = 923, diagnostic and antibody). Comparison between these groups, in terms of symptom prevalence, symptom trajectory, and disease duration is reported in the Results section.

**Table 1:** Testing status.

Type of SARS-CoV-2 Test*	Number of Respondents Tested	% of Respondents Tested**	Number of Respondents with Positive Results	% of Respondents with Positive Results**
Diagnostic (RT-PCR/antigen)	2330***	61.9%	600	15.9%
Antibody (IgG, IgM or both)	2166	57.6%	683	18.2%
Diagnostic (RT-PCR/antigen) or Antibody	3121	83.0%	1020	27.1%

*Some participants received both diagnostic (RT-PCR/antigen) and antibody tests. These participants are included in all rows of the table.

**Percentages are out of the total number of respondents (*N* = 3762).

***Total of 2362 received diagnostic tests, out of which 32 were inconclusive or awaiting response.

### 2.3 Outcomes

In this study we quantified disease duration, as well as symptom prevalence, probability time-course, severity, count, onset time, and temporal clustering. We also measured fatigue using the Fatigue Assessment Scale [17,18]. Return to baseline and working status were also measured.

The 203 symptoms (Appendix A) investigated were sourced from a combination of prior research, existing case-reports, literature review, and content shared by patients within support groups and on social media.

### 2.4 Statistics and data analysis

All statistics and data analysis were performed in MATLAB 2017a and 2020a, using a combination of built-in library functions and custom code.

#### 2.4.1 Survival function

**Fig. 1:**
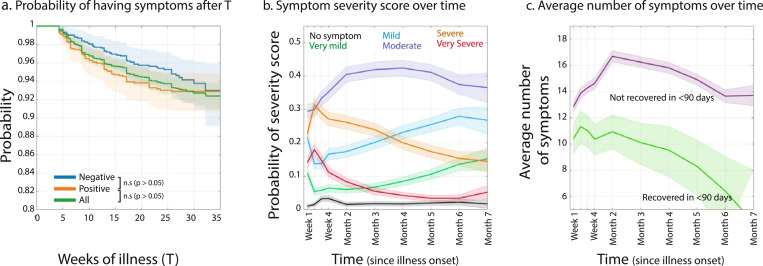
a) Survival function (Kaplan-Meier estimator), characterizing the distribution of disease duration for those who tested Negative (blue) on both diagnostic (RT-PCR/antigen) and antibody tests, those who tested Positive (orange) in either diagnostic or antibody test, and All (green) respondents. The Y axis indicates the probability that symptoms will persist longer than the time specified on the X axis. b) Probability of each symptom severity score over time. c) Average number of reported symptoms over time for those who recovered in less than 90 days (*n* = 154), or otherwise experienced symptoms for more than 90 days (*n* = 3505). a-c) In all plots, times are relative to initial symptom onset. Shaded regions represent 95% simultaneous confidence bands.

To investigate disease duration, the survey asked respondents to indicate the number of days their symptoms lasted. For non-recovered respondents, this number provided only a lower bound on the eventual duration of symptoms. To account for this censoring in the data, we characterized the distribution of durations using the Kaplan-Meier estimator[19]. The resulting survival function (Fig. 1a, Supplemental Figure S2a) measures the probability that symptoms will persist beyond any specified amount of time.

#### 2.4.2 Prevalence estimation

**Fig. 2:**
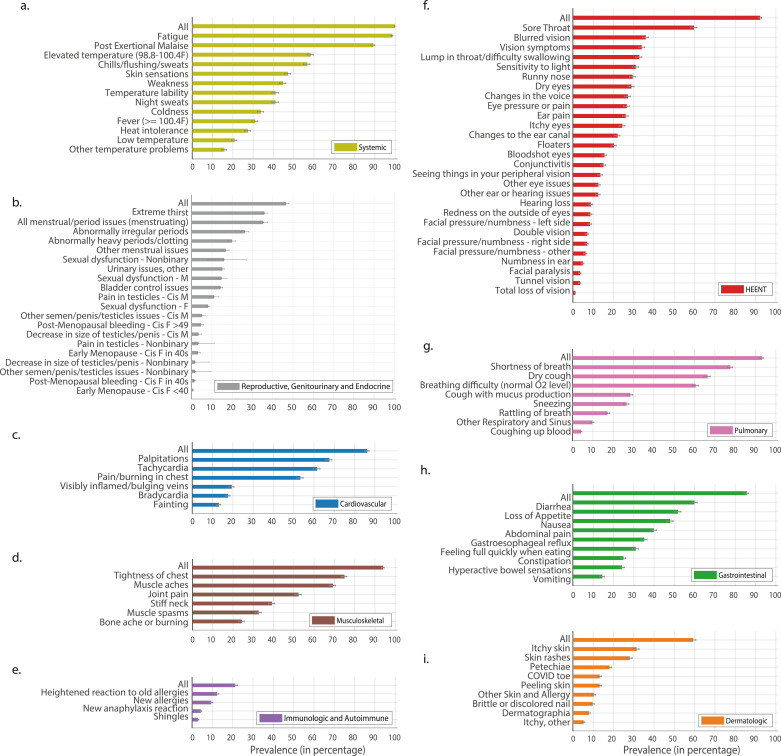
Symptom prevalence estimates (non-neuropsychiatric symptoms). Bars represent the percentage of respondents who experienced each symptom at any point in their illness. Symptoms are categorized by the affected organ systems. When all rows in a given panel use the same denominator, the first row, labeled “All,” indicates the percentage of respondents who experienced any symptoms in that category. Error bars are bootstrap 95% confidence intervals. In Fig. 2b, Sexual dysfunction is broken up into male (Sexual dysfunction - M) and female (Sexual dysfunction - F). “Cis M” refers to cisgender males, “Cis F” refers to cisgender females, and cisgender females are further broken down by age group: “Cis *F* <40″ indicates cisgender females age 39 or younger, “Cis F in 40s” indicates cisgender females age 40 to 49, and “Cis *F* >49″ indicates cisgender females age 50 or older.

**Fig. 3:**
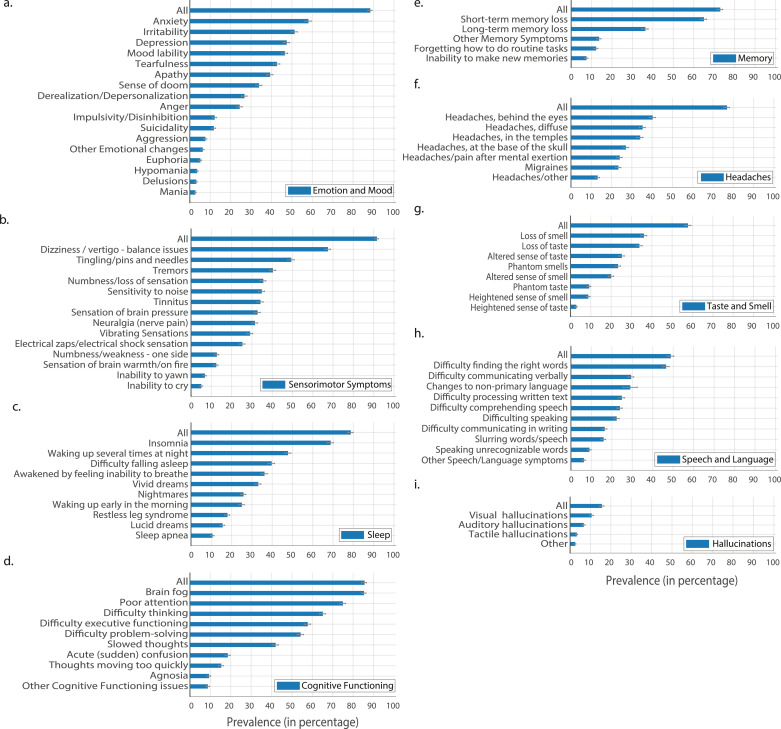
Symptom prevalence estimates for neuropsychiatric symptoms. Similar to Fig. 2, for neuropsychiatric symptoms, divided into nine sub-categories. Each bar represents the percentage of respondents who experienced that symptom. Error bars are bootstrap 95% confidence intervals.

**Fig. 4:**
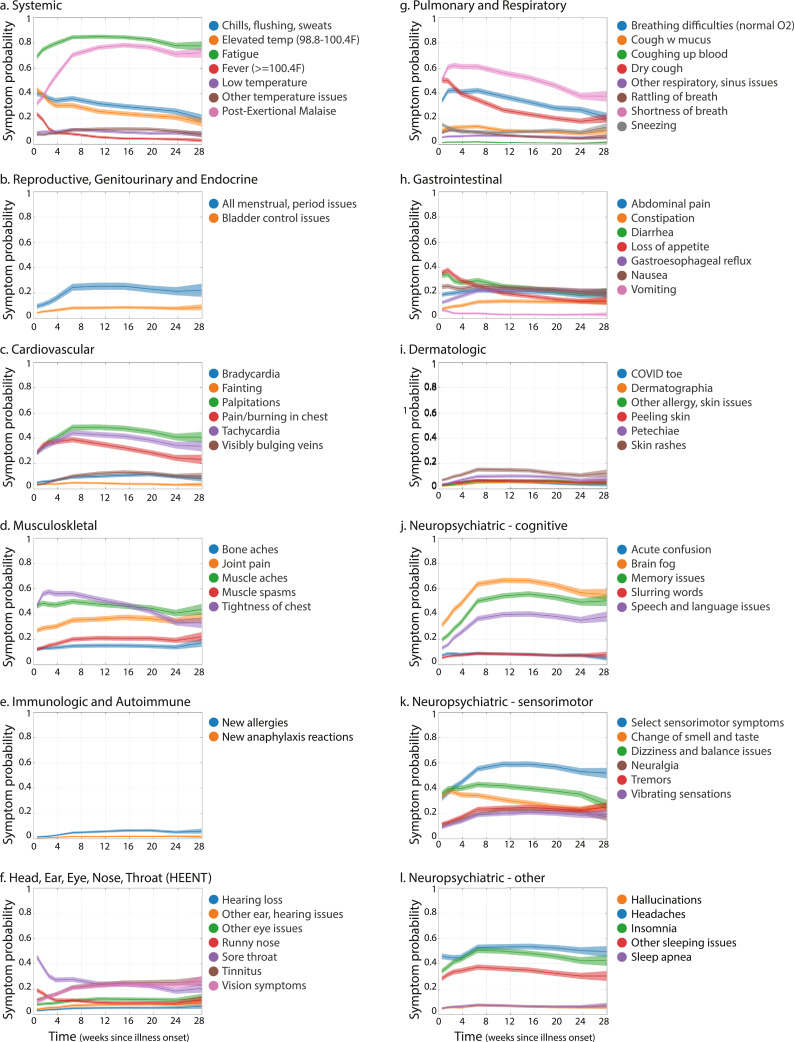
Symptom time courses. Plotted time courses represent the estimated probability of experiencing each symptom at each time point, given that recovery has not yet occurred (see Methods). Times are relative to initial illness onset. Symptoms are grouped according to the affected organ systems. Shaded regions show 95% simultaneous confidence bands, estimated separately for each symptom.

203 symptoms (Appendix A) were investigated by identifying their presence or absence. For 74 of these symptoms, respondents indicated at which points in their illness (weeks 1–4, months 2–7) they experienced the symptom, if at all. For each of the other 131 symptoms, participants indicated whether they had experienced the symptom at any point throughout the duration of their illness. Prevalence estimates were calculated by dividing the number of those who reported experiencing a symptom—either at a given time point (Fig. 4) or over the entire illness (Fig. 2, 3)—by the total number of participants to which the symptom applied (usually the whole sample, but occasionally out of a smaller population, such as respondents with menstrual cycles).

#### 2.4.3 Symptom time course estimation

The survey asked respondents to detail their experience of a subset of 74 symptoms over time. Eight symptoms were excluded from analysis, as their measurement required specialized equipment or tests that many participants may not have had access to (Appendix A, Figure S5). Respondents indicated whether each of these symptoms was present during a series of time intervals following the onset of their first symptoms (weeks 1–4, months 2–7). The time course of each symptom was defined as the probability of experiencing the symptom in each time interval, given that: 1) recovery had not occurred prior to the end of the interval, and 2) the symptom was applicable (menstruation-related symptoms are presented only for menstruating respondents). Plotted time courses in Fig. 4 are linearly interpolated between the centers of each time interval.

Fig. 5

**Fig. 5:**
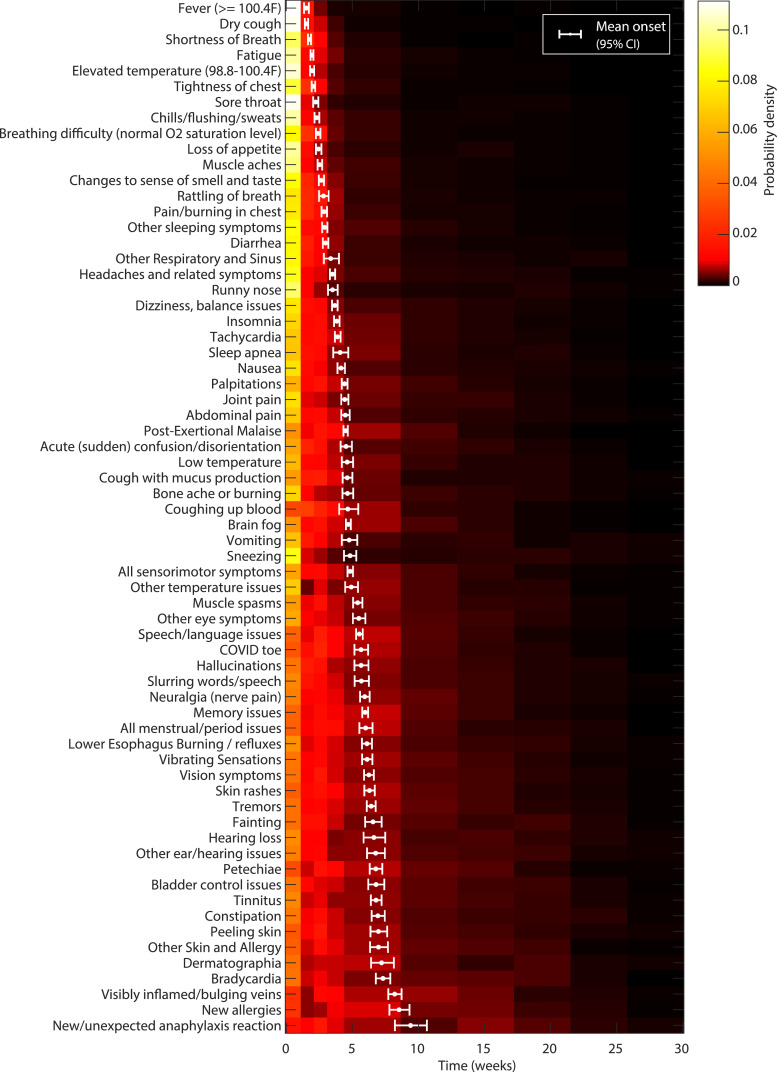
Symptom onset times. Heatmap shows the estimated probability distribution of the onset time for each symptom. White points and error bars show the mean onset time and 95% pointwise confidence intervals. Symptoms are sorted by mean onset time.

#### 2.4.4 Symptom severity and count

Overall symptom severity for each time interval (weeks 1–4, month 2–7) was measured using a Likert scale (no symptom, very mild, mild, moderate, severe, very severe).

#### 2.4.5 Symptom onset analysis

Continuous, piecewise-constant distributions were fit to onset times for each symptom using maximum likelihood and accounting for interval censoring (onset times for each respondent could only be measured up to the enclosing time intervals, described above). For each symptom, the estimated probability density at time *t* was given by the fraction of respondents who first experienced the symptom in the interval containing *t* (among those who experienced it at any point), divided by the duration of the interval. Mean onset time was calculated as the expected value of the estimated distribution.

#### 2.4.6 Symptom time course clustering

Symptom time courses were clustered using spherical k-means, a variant of k-means based on cosine distances [20]. Each time course is a 10-dimensional vector, representing the conditional probability of experiencing the symptom in each of the 10 time bins. The cosine distance is a monotonic function of the angle between vectors, and is insensitive to their magnitudes. Therefore, it is well suited to measuring differences between time course shapes (i.e. changes in relative amplitude over time), while remaining invariant to differences in overall symptom prevalence. We used a variant of Lloyd's algorithm designed for spherical k-means, with initialization by the k-means++ algorithm, and 100 random restarts to avoid suboptimal local minima. The number of clusters (*k* = 3) was chosen by hand, to provide a reasonable tradeoff between capturing structure in the data and obtaining a parsimonious explanation.

#### 2.4.7 Symptom time course sorting

**Fig. 6:**
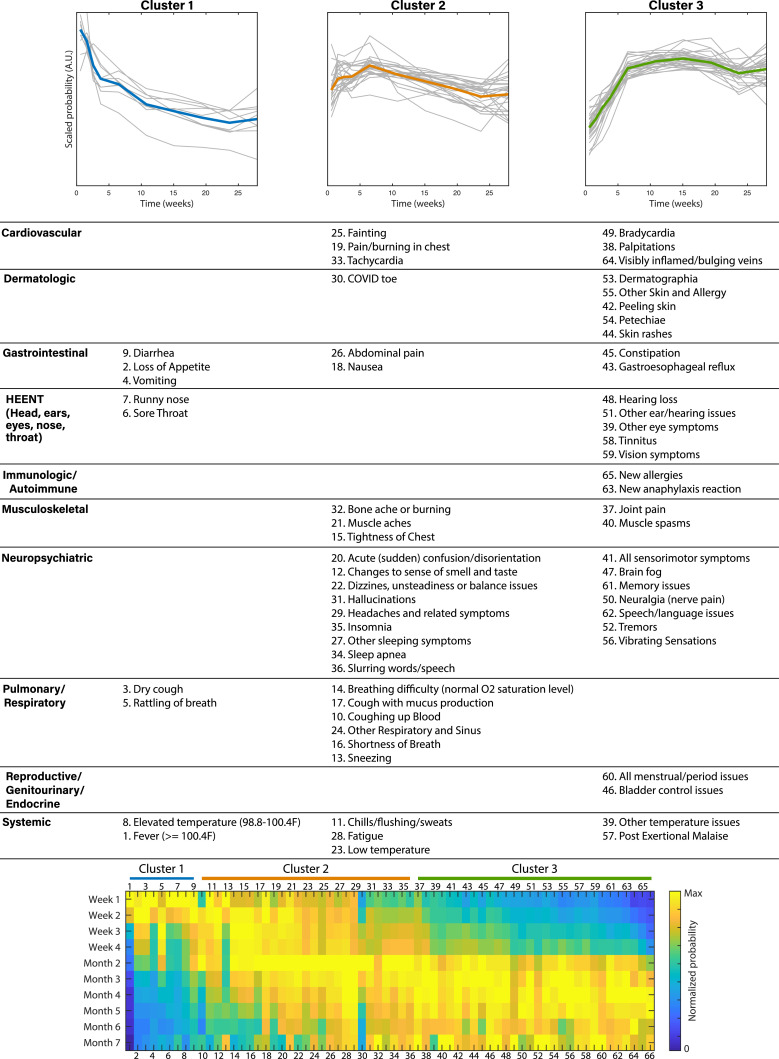
Symptom clusters, based on temporal similarities. Plots (top row) show time courses for the symptoms in each cluster (in gray) and their mean (Cluster 1 in blue, Cluster 2 in orange, Cluster 3 in green). Time courses have been scaled separately for each symptom (by root mean squared amplitude) to visually compare their shapes. The table lists symptoms in each cluster, grouped by the affected organ systems. The heatmap (bottom row) shows time courses for all symptoms, sorted such that similarly shaped time courses are adjacent (see Methods). Columns have been scaled by their maximum amplitudes for visual comparison. Symptoms are numbered according to their table entries.

The heatmaps in Fig. 6 and Figure S3 show normalized symptom time courses, sorted such that similarly-shaped time courses appear nearby in the ordering. To compute the sort ordering, similarity between time courses was measured using the cosine distance, as above. Classical multidimensional scaling (MDS) was then used to embed time courses into a one-dimensional Euclidean space, such that pairwise distances in the embedding space approximated the given cosine distances. Time courses were sorted according to their order in the embedding space.

#### 2.4.8 Confidence intervals

All confidence intervals and confidence bands were estimated using a nonparametric bootstrap approach with 10,000 iterations. Individual confidence intervals and pointwise confidence bands used the bias-corrected, accelerated (BCa) bootstrap[21]. Simultaneous confidence bands used the percentile bootstrap, with the percentile adjusted to give the correct simultaneous coverage probabilities.

#### 2.4.9 Fatigue assessment scale scores

Fatigue Assessment Scale (FAS) scores were calculated based upon participants’ subjective report during the “past one week.” The scores were summarized into three categories [17,18]: no fatigue (scores of 10–21), fatigue [22], [23], [24], [25], [26], [27], [28], [29], [30], [31], [32], [33], [34], and extreme fatigue (≥35).

See Appendix B for details of prevalence estimates, data stratification based on the diagnostic test time, and text analyses.

### 2.5 Role of funding

This study received no specific grant from any funding agency in the public, commercial, or not-for-profit sectors.

## 3 Results

### 3.1 Demographics

Survey response rate, calculated as the ratio of respondents who completed the survey [4] (918) to those who started the survey [7] (285), was 67.5%. This study included 3762 survey respondents based on the eligibility criteria described in Methods. Detailed demographics are listed in Table 2. The majority of respondents were women (78.9%, significantly more than other genders, *p* < 0.001, chi-squared test), white (85.3%, *p* < 0.001, chi-squared test), and between the ages of 30 and 60 (33.7% between ages 40–49, 27.1% ages 50–59, 26.1% ages 30–39). A total of 56 countries were represented in the sample. Most of the respondents resided in the United States (41.2%, *p* < 0.01, Tukey's HSD (honestly significant difference) multiple comparisons test). 91.9% of respondents completed the survey in English.

**Table 2:** Demographics of survey respondents.

Factor	Number of Respondents (*N* = 3762)	% of Respondents
Gender		
Women*	2969	78.9%
Men*	718	19.1%
Nonbinary	63	1.7%
Other	6	0.2%
Preferred not to answer	6	0.2%
Age Group (years)		
18–29	277	7.4%
30–39	905	24.1%
40–49	1166	31.0%
50–59	937	25.0%
60–69	380	10.1%
70–79	85	2.3%
80+	12	0.3%
Ancestry**		
White	3418	85.3%
Hispanic, Latino, Spanish Origin	150	3.7%
Asian, South Asian, SE Asian	134	3.3%
Black	80	2.0%
Middle Eastern, North African	66	1.7%
Indigenous Peoples	50	1.6%
Pacific Islander	3	0.1%
Other	98	2.5%
Prefer not to answer	9	0.2%
Environment		
Urban	1543	41.0%
Suburban	1586	42.2%
Rural	633	16.8%
Country of Residence		
USA	1567	41.2%
UK	1316	35.0%
France	163	4.3%
Canada	155	4.1%
Spain	99	2.6%
Netherlands	61	1.6%
Ireland	58	1.5%
Sweden	55	1.5%
Other	288	7.7%
Healthcare Worker		
Yes	668	17.8%
No	3094	82.2%
Hospitalization		
Non-Hospitalized and no visit to ER/Urgent Care	2133	56.7%
Visited ER or Urgent Care	1312	34.9%
Hospitalized	317	8.4%

*Respondents included 2961 (78.7%) cisgender women and 8 (0.2%) transgender women, 714 (19.0%) cisgender men and 4 (0.1%) transgender men.

**Respondents were invited to select multiple ancestries. Percentages in this section are thus based on the total number of ancestries reported. 182 (4.8%) respondents reported two ancestries, while 30 (0.8%) reported three or more ancestries.

More than half of respondents (56.7%, *p* < 0.001, chi-squared test) did not seek hospital-based care. 34.9% visited an ER or urgent care clinic but were not admitted to a hospital. 8.43% of respondents were hospitalized. 17.8% of respondents were healthcare workers (see Supplemental Material, Appendix C.2, for pre-existing conditions).

### 3.2 Symptoms and severity over time

#### 3.2.1 Symptom duration

Respondents were considered recovered if they identified themselves as no longer experiencing symptoms at the time of survey completion. 257 respondents (6.8%) recovered after day 28 of illness, and 3505 (93.2%) were still experiencing symptoms at the time of survey completion.

A survival function, measuring the probability that symptoms will persist beyond any specified amount of time (see Methods), is shown in Fig. 1a. In this Long COVID cohort, the probability of symptoms lasting beyond 35 weeks was 91.8% (95% confidence interval 89.5% to 93.5%), with no statistically significant difference between positively (diagnostic/antibody) and negatively tested groups (*p* = 0.18, chi-squared test), or men and women (*p* = 0.49, chi-squared test, Supplemental Figure S2a). Of the 3762 respondents, 2454 experienced symptoms for at least 180 days (six months). Among the remaining 1308 respondents, 233 recovered and the rest (*n* = 1075) took the survey before reaching six months of illness.

We described the Long COVID trajectory by assessing symptom severity and average number of symptoms over time. The probability of “severe” and “very severe” symptoms peaked during acute infection (<28 days), while the probability of “moderate” and “mild” rose gradually thereafter (Fig. 1b).

In those who recovered in less than 90 days, the average number of symptoms peaked at week 2 [mean number of symptoms (out of 66): 11.35, 95% confidence interval 13.58 to 9.44], and in those who did not recover in 90 days, the average number of symptoms peaked at month 2 (mean number of symptoms: 17.16, 17.78 to 16.54), with less decline over time (Fig. 1c, see Supplemental Figure S2 b-c for more comparisons between recovered and unrecovered participants). Respondents with symptoms for over six months experienced an average of 13.79 symptoms (95% confidence interval 12.68 to 14.88) in month 7.

#### 3.2.2 Symptoms experienced at any point

Overall symptom prevalence in 10 organ systems was estimated for a total of 203 symptoms (see Methods, Appendix A for list of symptoms). Table 3 summarizes these prevalence estimates for 18 categories (nine non-neuropsychiatric organ systems: systemic, reproductive/genitourinary/endocrine, cardiovascular, musculoskeletal, immunological and autoimmune, HEENT, pulmonary, gastrointestinal and dermatologic in Fig. 2, and nine neuropsychiatric sub-groups: cognitive dysfunction, speech and language, memory, headaches, smell and taste, sleep, emotion and mood, hallucinations, sensorimotor in Fig. 3, see Appendix F Table S6-S23 for raw data). Almost all participants experienced systemic (99.7%, 95% confidence interval 99.49% to 99.84%), and HEENT (100%) symptoms. Musculoskeletal, cardiovascular, gastrointestinal, pulmonary, and neuropsychiatric symptoms were prevalent in >85% of participants (further detail in Supplemental Tables S5-S21). The top three most debilitating symptoms listed by patients were: 1) fatigue (*n*>2652), 2) breathing issues (*n*>2242), and 3) cognitive dysfunction (*n*>1274). Participants experienced an average of 55.9+/- 25.5 (mean+/-STD) symptoms during their illness.

**Table 3:** Overall system prevalence data.

Symptom Category	Total #	Mean Prevalence	Lower CI	Upper CI
Systemic	3750	99.70	99.49	99.84
Reproductive / Genitourinary / Endocrine	2341	62.25	60.68	63.74
Cardiovascular	3236	86.04	84.90	87.16
Musculoskeletal	3530	93.85	93.03	94.60
Immunologic / Autoimmune	791	21.05	19.77	22.43
HEENT	3761	100.00	100.00	100.00
Pulmonary / Respiratory	3499	93.03	92.21	93.8
Gastrointestinal	3216	85.50	84.37	86.6
Dermatologic	2221	59.06	57.52	60.63
Neuropsychiatric - Cognitive Dysfunction	3212	85.43	84.29	86.55
Neuropsychiatric - Speech and Language	1828	48.62	47.00	50.21
Neuropsychiatric - Memory	2739	72.81	71.40	74.20
Neuropsychiatric - Headaches	2887	76.74	75.36	78.04
Neuropsychiatric - Smell and Taste	2166	57.60	56.06	59.21
Neuropsychiatric - Sleep	2955	78.58	77.25	79.88
Neuropsychiatric - Emotion and Mood	3320	88.25	87.19	89.26
Neuropsychiatric - Hallucinations	580	15.42	14.30	16.64
Neuropsychiatric - Sensorimotor	3440	91.44	90.48	92.29

#### 3.2.3 Symptoms over time

Symptoms exhibited varying time courses, defined as the probability of experiencing each symptom at each time point, given that recovery had not yet occurred (Fig. 4). Most symptoms had a prolonged probability of occurrence throughout the seven month period (see Appendix F Table S24 for the raw data; Supplemental Figure S9 for male vs. female comparison).

Symptoms were clustered in three groups (Fig. 6), according to the shapes of their time courses (i.e. changes in relative amplitude over time, ignoring their overall prevalence, see Methods). Cluster 1 consists of symptoms that are most likely to occur early in the illness, reaching a high point in the first two or three weeks, then decreasing in probability over time. Cluster 2 consists of symptoms with a relatively stable probability over time. Cluster 3 consists of symptoms most likely to increase sharply in the first two months. Their probability may plateau (like constipation), decrease slightly (like post-exertional malaise and fatigue), or increase slightly in the later months (like tinnitus, hearing loss, muscle spasms, and tremors). All clusters contained symptoms from multiple organ systems, and Cluster 3 contained symptoms from all but one organ system (pulmonary/respiratory symptoms). A general progression from early to late symptoms can also be seen in the heatmap of normalized time courses (Fig. 6 & Supplemental Figure S3), which have been sorted by similarity in shape (see Methods).

Symptom prevalence plots, together with the onset times and clusters (Fig. 2, Fig. 3, Fig. 4, Fig. 5, Fig. 6), show that symptoms spanned multiple organ systems. The mean number of organ systems affected in each respondent was 9.08 (95% confidence interval 9.04 to 9.13; see Symptom Details). Symptoms in the same organ-based category did not necessarily cluster together, and could appear across clusters. This indicates that symptoms affecting the same organ system can have differently shaped time courses and, conversely, symptoms affecting different organ systems can have similarly shaped time courses. Systemic and neurological/cognitive symptoms were the most likely to persist from disease onset to month 7 (see Symptom Details).

### 3.3 Neuropsychiatric symptoms

#### 3.3.1 Brain fog/Cognitive dysfunction and memory impairment

85.1% (95% confidence interval 83.9% to 86.2%) of respondents (3203) reported experiencing brain fog and cognitive dysfunction, including poor attention, executive functioning, problem solving, and decision making (Fig. 3d, Supplemental Table S15 for prevalence of sub-symptoms). 72.8% (71.4% to 74.2%) of all respondents (2739) experienced memory impairments, including both short-term and long-term memory loss (Fig. 3e & Supplemental Table S16 for prevalence of sub-symptoms).

For 31.2% (29.7% to 32.7%) of respondents, the onset of brain fog/cognitive dysfunction occurred in the first week of symptoms. Reports of cognitive dysfunction increased over the first three months, peaking at 66.7% (65.1% to 68.2%), then decreased slightly in the following months. 55.5% (52.5% to 58.8%) of month 7 respondents experienced cognitive dysfunction during month 7 (Fig. 4j).

The probability of experiencing memory symptoms increased the first few months, with 55.9% (54.3% to 57.5%) reporting memory symptoms in month 4. 50.5% (47.3% to 53.6%) of respondents with symptoms for over six months experienced memory symptoms in month 7 (also Fig. 4j).

Of those who experienced memory and/or cognitive dysfunction symptoms and had a brain MRI, 87% of the brain MRIs (*n* = 345, of 397 who were tested) showed no abnormalities.

##### 3.3.1.1 Impact of cognitive dysfunction/memory on daily abilities and impact by age

**Fig. 7:**
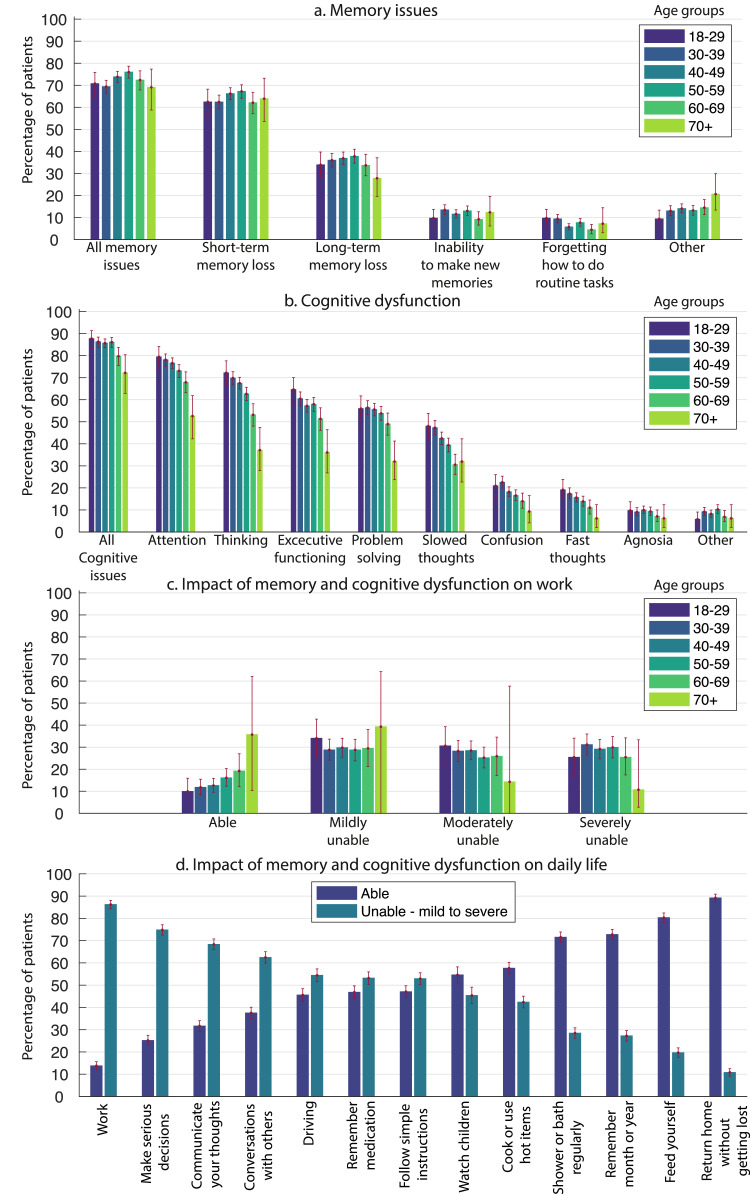
Memory and cognitive dysfunction. a) Percentage of respondents in six age groups who experienced different types of memory impairments. b) Same as (a) for cognitive dysfunction. c) Impact of memory and cognitive dysfunction on work (for those who work), for different age groups. Participants were asked to rate the impact by choosing one of the four options “Able, Mildly unable, Moderately unable, and Severely unable”. d) Overall impact of memory and cognitive dysfunction on daily life. Participants to whom the question was not applicable were excluded. Error bars show bootstrap 95% confidence intervals.

88.0% of the total respondents (3310) experienced cognitive dysfunction, memory loss, or both at similar rates across all age groups (Fig. 7a-c). The greatest area of impact reported was on work, with 86.2% (95% confidence interval 84.4 to 88.0%) of working respondents feeling mildly to severely unable to work (see Impact on Work section below for a discussion of the working status of respondents). See Fig. 7d for the detailed list of memory and cognitive dysfunction impacts on daily life.

Selected quotes from respondents who described specific instances of memory loss or brain fog can be found in Appendix D.

#### 3.3.2 Other neuropsychiatric symptoms

The most common neuropsychiatric category was sensorimotor symptoms (prevalence of 91.44%, 95% confidence interval 90.48% to 92.29%). Other neuropsychiatric symptoms included speech and language issues (48.6%, 47.0% to 50.2%), headaches (77.0%, 75.4% to 78.0%), emotion and mood (88.3%, 87.2% to 89.3%), taste and smell (57.6%, 56.0% to 59.2%), and hallucinations (23.2%, 21.9% to 24.6%). See Supplementary Material for detailed discussion of all sub-symptoms.

78.6% (95% confidence interval 84.0% to 79.9%) of respondents experienced difficulty with sleep (Fig. 3c, Supplemental Table S19 for full prevalence data). Table 5 lists the prevalence of each sleep symptom, as well as the percentage of respondents with that symptom who also reported it as pre-existing (before COVID-19 infection).

**Table 5:** Prevalence of sleep issues before and during illness.

Sleep Symptom	Experienced During Illness (of all participants)	Had Symptom Before Illness (of those who experienced the symptom)
Insomnia	60% (67.1 to 70.1%)	21%
Night Sweats	41% (39.2 to 42.4%)	16%
Awakened Feeling Unable to Breathe	36% (34.5 to 37.6%)	N/A
Restless Legs	18% (16.6 to 19%)	14%
Sleep Apnea	10% (9.5 to 12.8%)	34%
Vivid Dreams	33% (31.5 to 34.5%)	23%
Nightmares	26% (24.3 to 27.1%)	20%
Lucid dreams	15% (14.2 to 16.6%)	34%

### 3.4 Special considerations

Nearly half of respondents (43.4%) were diagnosed with at least one condition post-acute COVID-19 infection (see Table S2 Appendix C.3).

#### 3.4.1 Postural orthostatic tachycardia syndrome (POTS)

Of the 2308 patients who reported tachycardia, 72.8% (*n* = 1680) reported being able to measure their heart rate in standing vs. sitting posture. Of those, 30.65% (*n* = 515) reported an increase in heart rate of at least 30 BPM on standing, suggesting the possibility of Postural Orthostatic Tachycardia Syndrome (POTS, [22])

#### 3.4.2 Reactivation and test results for latent disease

Since being infected with SARS-CoV-2, 2.8% (95% confidence interval 2.3% to 3.3%) of respondents reported experiencing shingles (varicella zoster reactivation), 6.9% reported current/recent Epstein–Barr virus (EBV) infection, 1.7% reported current/recent Lyme infection, and 1.4% reported current/recent Cytomegalovirus (CMV) infection. Detailed results are shown in Table 6.

**Table 6:** Test results for latent disease.

Virus	Positive*	Positive (past)	Negative	Total Tested
Epstein-Barr (EBV)	40	309	231	580
Lyme Disease	7	34	366	407
Cytomegalovirus (CMV)	4	85	204	293

* Includes both current and recent cases.

#### 3.4.3 Post-Exertional malaise (PEM)

**Fig. 8:**
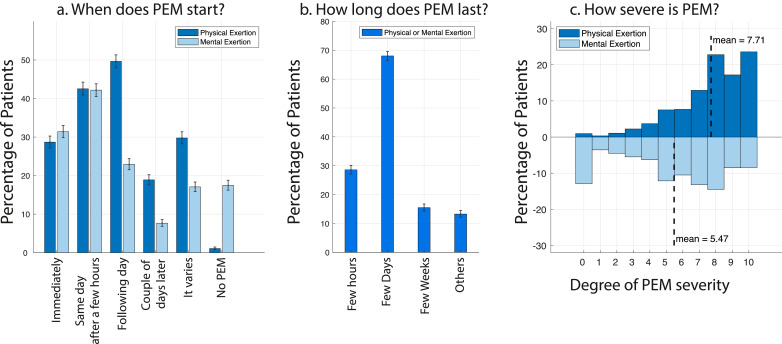
Worsening or relapse of symptoms after physical or mental activity (post-exertional malaise). When does it start (a), how long does it last (b), and how severe is it? (c) (all patients who experienced PEM, *n* = 3350). Error bars are bootstrap 95% confidence intervals.

The survey asked participants whether they have experienced “worsening or relapse of symptoms after physical or mental activity during COVID-19 recovery”[23]. Borrowing from Myalgic Encephalomyelitis/Chronic Fatigue Syndrome (ME/CFS) terminology [24], this is referred to as post-exertional malaise (PEM). 89.1% of participants (95% confidence interval 88.0% to 90.0%) reported experiencing either physical or mental PEM. PEM was triggered at various time points after exertion, (Fig. 8a) and, for the majority of respondents, lasted for a few days (68.3%, 66.4% to 69.6%, Fig. 8b). The distribution of severity scores (out of 10) is shown in Fig. 8c.

### 3.5 Symptoms by test result

Among respondents who received a diagnostic test (RT-PCR or antigen) for SARS-CoV-2 at any point during their illness, 1730 tested negative and 600 tested positive. The primary difference between these two groups was the time elapsed between symptom onset and testing, with a median of 6 days for those who tested positive and 43 days for those who tested negative (*p* < 0.001, Mann-Whitney U test) (Supplemental Figure S6). Symptoms were remarkably similar between the two groups. We compared symptom prevalence among positively and negatively tested respondents, stratified by test time. Out of 203 symptoms, 203 showed no statistically significant difference (*p* > 0.05; Fisher's exact test, Bonferroni corrected). The loss of smell and taste were the only exceptions (loss of smell: 22.2% (negative) vs 60.8% (positive), *p* < 0.0001; 21.5% loss of taste: 21.5% (negative) vs. 54.9% (positive), *p* < 0.0001; Fisher's exact test, Bonferroni corrected). In addition, 683 participants tested positive for SARS-CoV-2 antibodies (either IgG, IgM, or both).

**Fig. 9:**
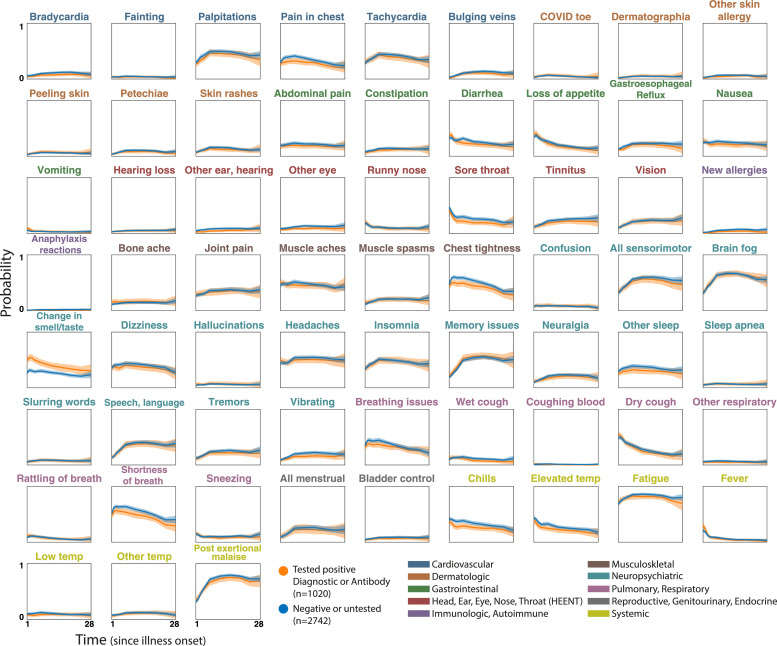
Symptom time courses for participants with COVID-19 confirmed via testing vs. rest of the population. Plots show symptom time courses (similar to Fig. 4) for respondents who were confirmed COVID-positive via diagnostic or antibody testing (orange) vs those without a positive confirmation (untested or tested negative, blue). Shaded regions show simultaneous 95% confidence bands. Symptom names are colored according to the affected organ systems.

Furthermore, respondents experienced similar variation in symptoms over time, despite differences in testing status. For 65 out of 66 symptoms, time courses overlapped substantially between participants with confirmed COVID-19 (*n* = 1020, positive RT-PCR, antigen, or antibody test at any point) and participants with no positive test result (*n* = 2742, Fig. 9). As above, change in smell/taste was the lone exception. Similar overlap was observed when separately comparing positively tested participants to negatively tested and untested participants (Supplemental Figures S7 and S8).

### 3.6 Recovery, return to baseline

#### 3.6.1 Relapses: triggers & experience

**Fig. 10:**
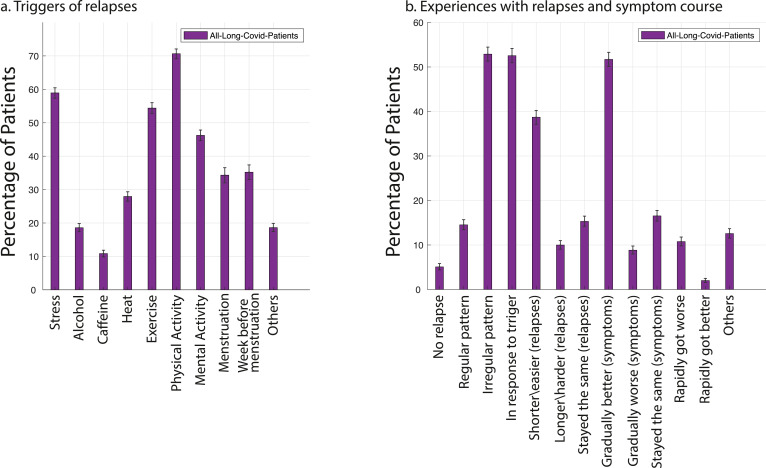
Triggers and experience of relapses. a. Triggers for relapses/worsening of symptoms b. Experience of symptoms over time and relapses. Error bars are bootstrap 95% confidence intervals.

Patients with Long COVID can experience relapsing-remitting symptoms[7]. In this cohort, a minimum of 85.9% (84.8% to 87.0%) of respondents reported experiencing relapses (Fig. 10a-b). Respondents characterized their relapses as occurring in an irregular pattern (52.8%, 95% confidence interval 51.2% to 54.4%) and in response to a specific trigger (52.4%, 50.8% to 54.0%). The most commonly reported triggers of relapses (or of general worsening of symptoms) were physical activity (70.7%, 69.2% to 72.1%), stress (58.9%, 57.3% to 60.5%), exercise (54.39%, 52.8% to 56.0%), and mental activity (46.2%, 44.7% to 47.8%). More than a third of menstruating participants experienced relapses during (34.3%, 32.0% to 36.5%) or before menstruation (35.2%, 33.0% to 37.3%).

#### 3.6.2 Remaining symptoms after 6 months

**Fig. 11:**
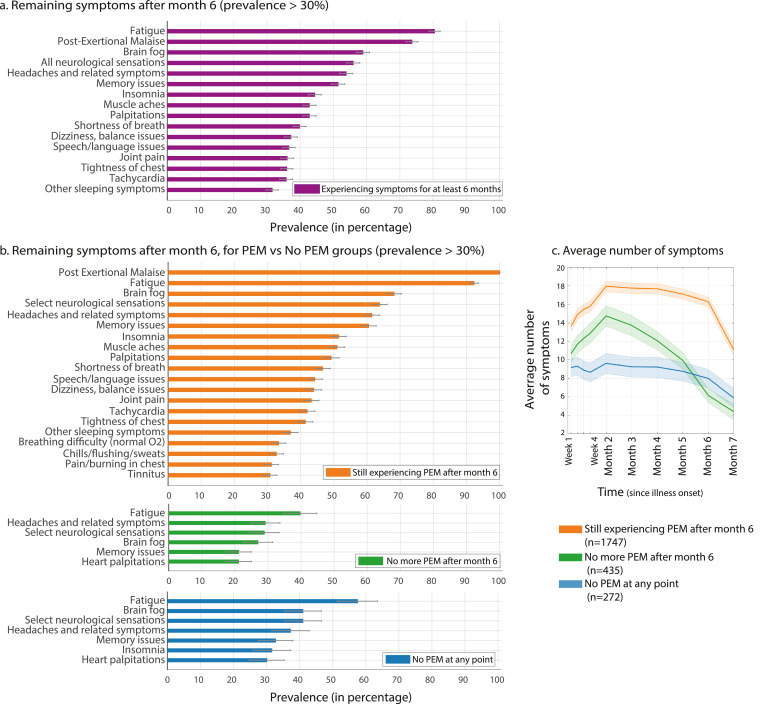
Remaining symptoms after six months. a) Symptoms remaining after six months. b) Symptoms remaining after six months for respondents still experiencing PEM after six months (orange), respondents not experiencing PEM after six months (green), and respondents who never experienced PEM (blue). c) Average number of symptoms over time for each group in (b). Error bars are bootstrap 95% confidence intervals.

Only 164 out of 3762 participants (4.4%) experienced a temporary break in symptoms (Supplemental Figure S4). The remaining participants reported symptoms continuously, until symptom resolution or up to taking the survey. A total of 2454 (65.2%) respondents experienced symptoms for at least six months. For this population, the top remaining symptoms after six months were primarily a combination of systemic and neurological symptoms (Fig. 11a), including fatigue (80.0%, 95% confidence interval 78.5% to 81.6%), post-exertional malaise (73.3%, 71.5% to 75.1%), cognitive dysfunction (58.4%, 56.5% to 60.2%), sensorimotor symptoms (55.7%, 53.7% to 57.6%), headaches (53.6%, 51.5% to 55.5%), and memory issues (51.0%, 49.1% to 53.0%). Respondents who still experienced PEM after six months had significantly more symptoms than those who never experienced PEM, and those whose PEM resolved by month 6 (Fig. 11b, c).

#### 3.6.3 Fatigue assessment

**Fig. 12:**
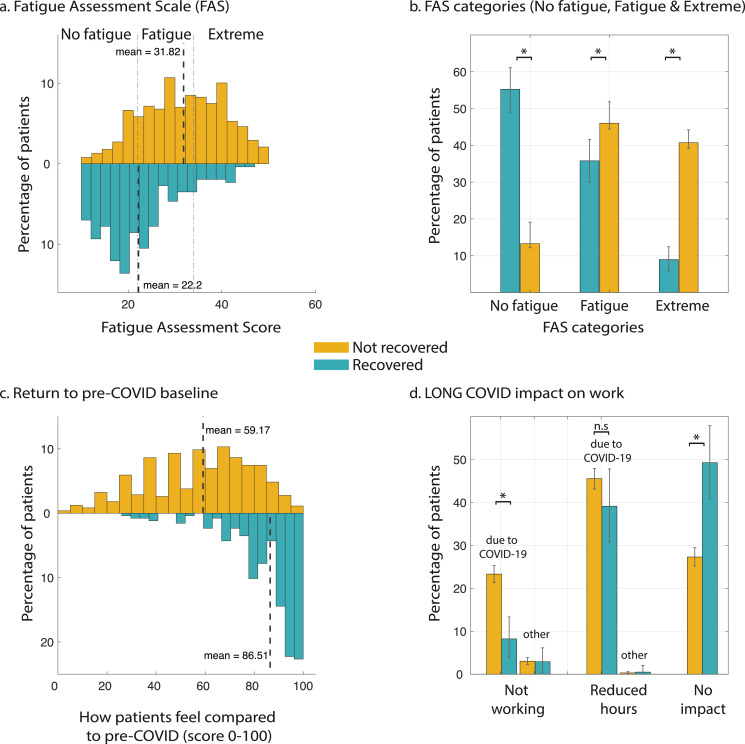
Return to baseline and work impact. a) Distribution of Fatigue Assessment Scale scores for recovered (*n* = 257, blue) and unrecovered (*n* = 3505, yellow) population. The vertical dashed lines indicate the range for “No fatigue”[10], [11], [12], [13], [14], [15], [16], [17], [18], [19], [20], [21], “Fatigue”[22], [23], [24], [25], [26], [27], [28], [29], [30], [31], [32], [33], [34], and “Extreme” (>=35). Mean values for each distribution are also marked. b) Percentage of participants in each of the three categories. c) Distribution of scores in response to “return to pre-COVID” health baseline, where 0 indicates worst (most different from baseline) and 100 indicates best (most similar to baseline). d) Working status due to COVID-19. Error bars show 95% simultaneous confidence interval.

We contrasted the Fatigue Assessment Scale (FAS) scores[17,18] of unrecovered (*n* = 3505, experiencing symptoms for average of 144 days) and recovered participants (*n* = 257, experiencing symptoms for average of 91 days). On average, unrecovered participants had higher FAS scores than recovered participants (31.8 vs 22.2, *P* < 0.001, Mann-Whitney *U* test, Fig. 12a). 55.3% (95% confidence interval 49.4% to 61.5%) of recovered participants were classified as having no fatigue. This is significantly more than the 13.2% (12.2% to 14.4%, *P* < 0.001, Mann-Whitney *U* test, Fig. 12b) of unrecovered participants who experienced no fatigue at the time of survey. 40.7% (39.9% to 42.3%) of unrecovered participants were classified as experiencing extreme levels of fatigue, which was significantly higher than the 8.9% (5.8% to 12.8%) of recovered participants in this category (*P* < 0.001, Mann-Whitney *U* test, Fig. 12b).

#### 3.6.4 Return to baseline

Respondents were asked, *“How would you rate how you feel today, on a scale of 0–100% (with 100% being your pre-COVID baseline)?”* (Fig. 12c). Unrecovered participants reported a mean score of 59.2, while recovered participants reported a mean score of 86.5 (*p*<0.001, Mann-Whitney *U* test). 23.1% of respondents considered "pacing" to be "significantly helpful" (out of 1788 who tried it)—a greater fraction than for other treatments reported. 18.8% found it “slightly helpful”.

#### 3.6.5 Impact on work

Among unrecovered respondents who worked before becoming ill, only 27.3% (95% confidence interval 25.3% to 29.4%) were working as many hours as they were prior to becoming ill at the time of survey, compared to 49.3% (40.8% to 57.9%) of recovered respondents (see Fig. 12d). Nearly half (45.6%, 43.2% to 48.0%) of unrecovered respondents were working reduced hours at the time of the survey, and 23.3% (21.3% to 25.4%) were not working at the time of the survey as a direct result of their illness. This included being on sick leave, disability leave, being fired, quitting, and being unable to find a job that would accommodate them. The remaining respondents retired, were volunteers, or did not provide enough information to determine their working status. Overall, 45.2% (42.9% to 47.2%) of respondents reported requiring a reduced work schedule compared to pre-illness. 22.3% (20.5% to 24.3%) were not working at the time of survey due to their health condition. See Appendix B for thematic analysis of participants’ free text responses [25], [26], [27] on their working status (selected quotes in Appendix D).

## 4 Discussion

Results from this international online survey of 3762 individuals with suspected or confirmed COVID-19 illness suggest that Long COVID is composed of heterogeneous sequelae that often affect multiple organ systems, with impact on functioning and ability to work. To our knowledge, this represents the largest collection of symptoms identified in the Long COVID population to date. While several others have investigated Long COVID symptoms [8,28], our approach also allowed for the first representation of individual symptom trajectories over time.

Our analyses show that participants experience symptoms that are not commonly mentioned in public discussion of Long COVID[3,29,30], and may benefit from further research. These include but are not limited to: anaphylaxis and new allergies, seizures, suicidality, changes in sensitivity to medication, vision loss, hearing loss, and facial paralysis. Several of these symptoms (e.g. anaphylaxis,new allergies, changes in sensitivity to medications), as well as the more commonly reported Long COVID symptoms (e.g. dizziness and tachycardia), overlap with symptoms of Mast Cell Activation Syndrome (MCAS), possibly warranting further exploration into the role of mast cells in Long COVID [31].

Dysautonomia, including Postural Orthostatic Tachycardia Syndrome (POTS), and Myalgic Encephalomyelitis/Chronic Fatigue Syndrome (ME/CFS) appear as highly possible diagnoses for this population [32]. By the time respondents took the survey, 155 had received a diagnosis of POTS, and 118 had received a diagnosis of ME/CFS. 33.9% of respondents who reported tachycardia measured an increase of at least 30 BPM when standing, suggesting a possible POTS diagnosis [33]. Given these findings, we suggest that all patients who present with any signs or symptoms of POTS, including tachycardia, dizziness, brain fog, or fatigue, be screened for POTS [22].

To investigate the possible overlap with ME/CFS in this population, we asked participants to identify whether they experienced worsening of symptoms after physical or mental exertion. This is a phenomenon known as Post-Exertional Malaise (PEM), which is one of the three required symptoms for ME/CFS diagnosis along with unrefreshing sleep and a reduction in ability to engage in pre-illness levels of activity [34]. We found PEM to be highly represented in this cohort (89.1% at any time during the course of illness, 72.2% at month 7). Intriguingly, among those still experiencing symptoms at month 6 with no PEM (*n* = 707, 28.8%), fatigue was still the most common symptom.

This work highlights the wide range of neurologic symptoms experienced by patients with Long COVID. Prior studies have identified evidence of cognitive dysfunction induced by COVID-19 illness, with few studies in the non-hospitalized population [32,35]. Memory and cognitive dysfunction, experienced by over 88% of respondents, were the most pervasive and persisting neurologic symptoms in this cohort, equally common across all ages, and with substantial impact on work and daily life. Memory and cognitive dysfunction, together with other commonly reported neuropsychiatric symptoms, may point to larger neurological issues involving both the central and peripheral nervous system.

The reduced work capacity because of cognitive dysfunction, in addition to other debilitating symptoms, translated into the loss of hours, jobs, and ability to work relative to pre-illness levels. Additionally, only 55.3% of recovered respondents had Fatigue Assessment Scores ranked as “no fatigue”. This could suggest that some respondents who reported that they were no longer experiencing symptoms considered any lingering effects as part of their new health baseline. For those who returned to their job, respondents reported experiencing relapses triggered by the mental exertion and stress of work, often needing to go back on leave. This emphasizes the importance of all patients having adequate time off to recover, being able to qualify for disability benefits if long-term assistance is needed, and receiving accommodations at work including telecommuting, flexible hours, and phased returns. Lower wage earners may find it especially challenging to access accommodations and benefits, yet they are in need of protections the most to ensure financial stability [36]. Further investigation could be done to measure the quality of life after Long COVID across socioeconomic strata.

Overall, these findings suggest that the morbidity of COVID-19 illness has been greatly overlooked. Patients experience multisystem symptoms for over seven months, resulting in significant impact to their lives and livelihoods.

Our analysis confirms prior findings that, with the exception of change to smell and taste, symptoms are not significantly different between those who test positive for SARS-CoV-2 and those who test negative (or have not been tested), but who otherwise show strongly suggestive symptoms [7,37]. The sensitivity of diagnostic tests may depend on the primer/probe sets [38,39]. Furthermore, the likelihood of false negatives increases after day 3 of symptom onset, when the false negative rate is 20%, reaching 66% by day 21[40]. This reinforces the need for early testing in patients with suspected SARS-CoV-2 infection, given that up to 54% of patients could have an initial RT-PCR false-negative result [41]. The importance of early testing was reflected in this cohort as well: the median number of days between first experiencing symptoms and being tested was 6 days for those who tested positive and 43 days for those who tested negative. Access to adequate diagnostic tests in the early stages of the pandemic was notably limited, which likely contributed to respondents in this cohort being unable to be tested and/or being tested later in their illness [42]. The site of sample collection, e.g. nasopharyngeal swab sampling vs. sputum testing [43], or stool vs. respiratory specimens [44] can also play an important role in testing accuracy [43]. Regarding antibody testing, it has been reported that antibody levels decrease with time [44,45], that males are likely to retain antibodies longer than females [46], and that antibody tests can be less accurate for females [47]. These results may be relevant to our cohort, of whom the majority was female. There is also evidence that patients with neurological symptoms but minimal respiratory symptoms may fail to seroconvert [33]. Together, these findings indicate that absent or negative SARS-CoV-2 diagnostic and antibody tests should not be used as an indicator to rule out Long COVID in patients who otherwise have suggestive symptoms [37,[48], [49], [50]]. Further investigation is needed to understand why some Long COVID patients test positive and others do not, despite having similar symptom courses.

While the majority of participants did not report receiving a positive SARS-CoV-2 diagnostic or antibody test result, our analysis of symptoms in confirmed and suspected COVID-19 groups indicates that this is only a limitation in the sense that diagnostic serology is lacking. Removing suspected COVID-19 participants from our analysis does not change the results.

The retrospective nature of the study exposes the possibility of recall bias, which could impact the reliability of symptom prevalence estimates. Because participants were asked to report any symptoms experienced within the designated time periods, both overreporting and underreporting of symptoms are possible. As the survey was distributed in online support groups, there exists a sampling bias toward Long COVID patients who joined support groups and were active participants of the groups at the time the survey was published. The effort to complete the survey may have deterred some respondents who experienced cognitive dysfunction, or were no longer ill and did not have incentives to participate. Furthermore, most respondents (91.6%) had not been hospitalized. The severity of illness that the survey captured may not be representative of the average Long COVID patient because of these issues. Additionally, despite eight translations and inclusive outreach efforts, the demographics were strongly skewed towards English speaking (91.9%), white (85.3%), and higher socioeconomic status (see Figure S1). Moreover, the study required respondents to have stable internet and email addresses, which may have excluded participants who lacked access and/or had low digital literacy. In future studies, more outreach and partnerships with diverse groups, low-income communities, and communities of color can be established to counter sampling bias.

As a result of the above limitations, the study may not be representative of the entire Long COVID population or their experiences.

We suggest that the results laid forth be considered only in the context of this study; extrapolation of the results to all patients with Long COVID requires caution.

## Declaration of Competing Interest

All authors have completed the ICMJE uniform disclosure form and declare: no support from any organization for the submitted work. All authors except HED and GSA declare no financial relationships with any organization that might have an interest in the submitted work in the previous three years, no other relationships or activities that could appear to have influenced the submitted work. HED reports personal fees ($500 speaking fee) from Council for Medical Specialty Society, outside the submitted work. GSA reports personal fees ($1000 speaking fee) from Council for Medical Specialty Society and Karolinska Institute, outside the submitted work.

## Appendix Supplementary materials

Image, application 1
