# Supplementary material for: Characterizing long COVID in an international cohort: 7 months of symptoms and their impact
Source: eClinicalMedicine. 2021 Jul 15;38:101019. doi: 10.1016/j.eclinm.2021.101019 (PMC8280690; doi:10.1016/j.eclinm.2021.101019)
Supplement: Supplementary file 1 [file mmc1.docx]

# **Supplemental Material**

# Appendix A: Symptom information

## *A.1 Symptom categories*

Symptoms are grouped in 10 categories, as below, given which organ/system they manifest in:

- Systemic: fatigue, temperature, weakness, flushing and sweating related symptoms, and post-exertional malaise
- Neuropsychiatric - because of the number and prevalence of neuropsychiatric symptoms assessed, they are broken into the following nine sub-categories and discussed separately:
- Cognitive Functioning
- Memory
- Speech and Language
- Sensorimotor Symptoms
- Sleep
- Headaches
- Emotion and Mood
- Taste and Smell
- Hallucinations
- Cardiovascular: heart rate, palpitations, blood pressure (excluded from in-depth analysis, see Supplemental Figure S5), visibly bulging veins, clots, and pain/burning in the chest
- Dermatologic: itchiness, rashes, and obvious changes in skin and nails
- Gastrointestinal: GI upset, hyperactive bowel sensations, and appetite-related symptoms
- Pulmonary and Respiratory - encompasses breathing, coughing, and sneezing, and oxygen saturation related symptoms (excluded from in-depth analysis, see Supplemental Figure S5)
- Head, Ear, Eye, Nose, Throat (HEENT): both physical and sensory symptoms related to the eyes, ears, nose, mouth, throat, and face including facial paralysis and numbness. Headaches are captured in the Neuropsychiatric category.
- Reproductive, Genitourinary, and Endocrine: symptoms related to menstruation and lack thereof, symptoms related to male reproductive function, symptoms related to sexual function, symptoms related to thirst and urinary function, and low and high blood sugar (excluded from in-depth analysis, see Supplemental Figure S5)
- Immunologic and Autoimmune: new and heightened immune responses
- Musculoskeletal: chest tightness and aches and pain throughout the musculoskeletal system

## *A.2 Symptom list*

Total of eight symptoms form the following list were excluded from our analyses, as their measurement required specialized equipment or tests that many participants may not have had access to. Excluded symptoms included 1. high blood pressure, 2. low blood pressure, 3. thrombosis, 4. confirmed seizures, 5. suspected seizures, 6. low oxygen levels, 7. high blood sugar, and 8. low blood sugar.

**Non-neuropsychiatric symptoms**


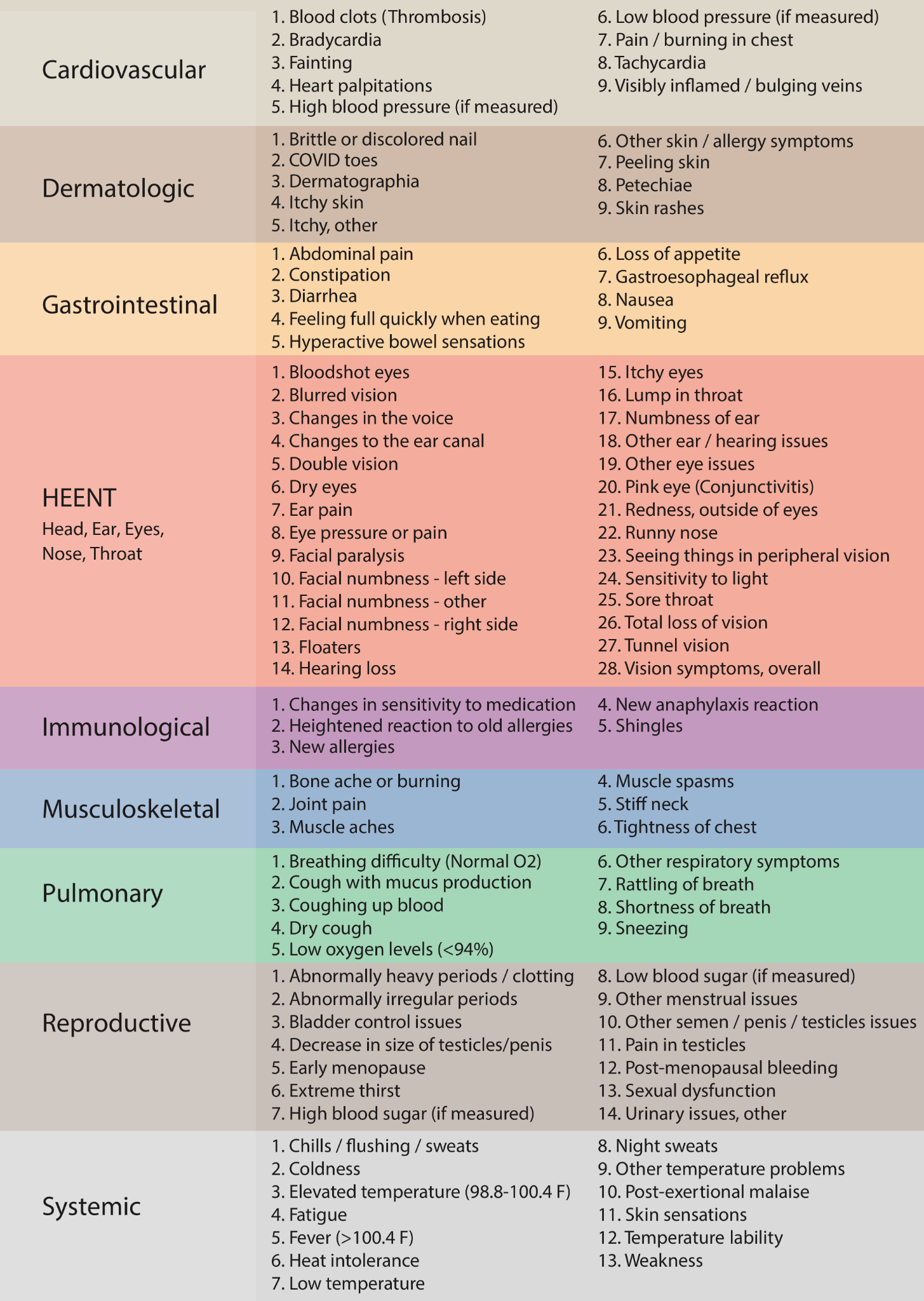


**Neuropsychiatric symptoms**


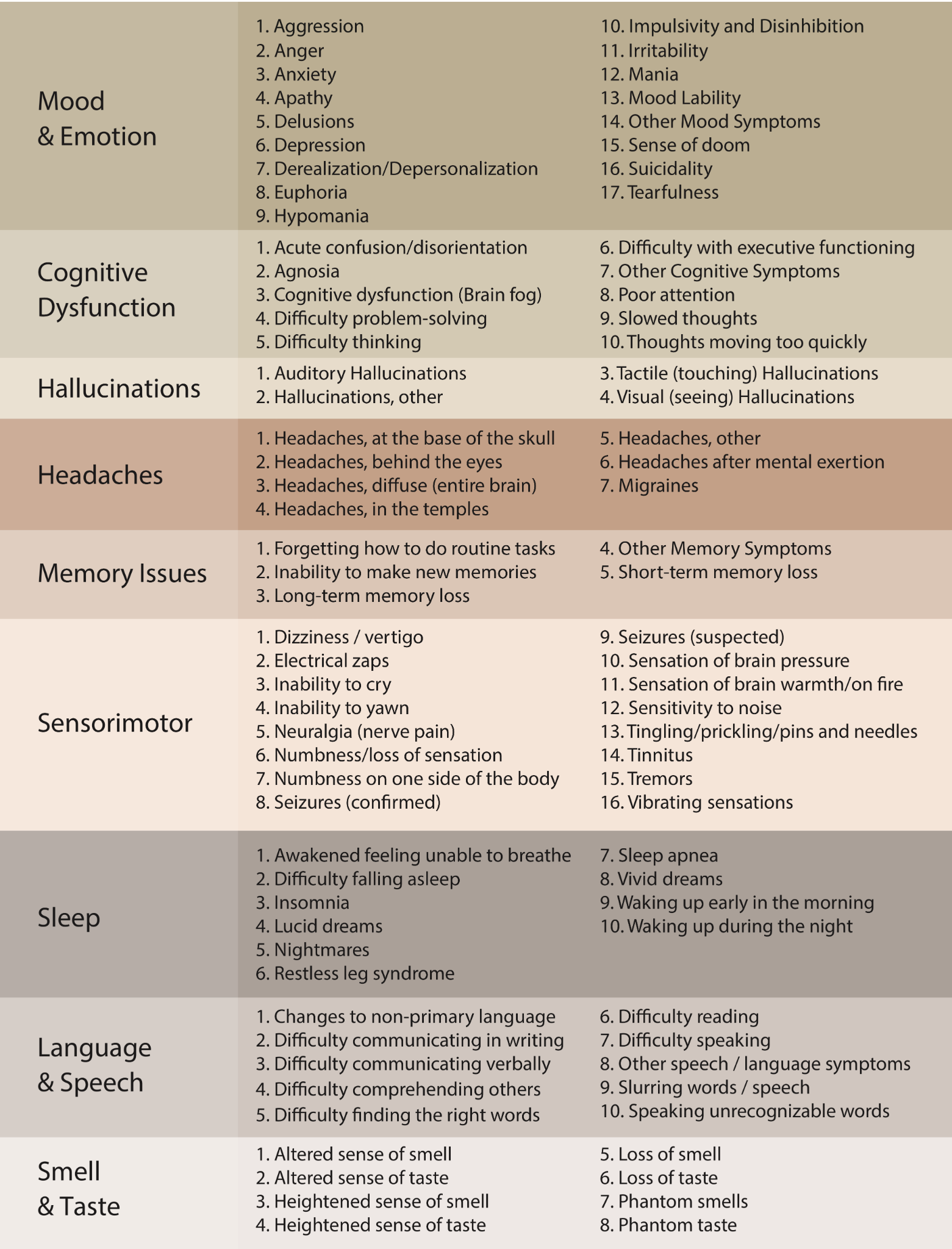


##

## *A.3 Sensorimotor symptoms - impacted body parts* For each of these symptoms, we asked participants to write in the part(s) of the body that was affected, and performed natural language processing to identify the top four locations affected for each symptom.

*Table S1. Top 4 affected body parts of sensorimotor symptoms*

| **Symptom** | **Top location** | **2nd location** | **3rd location** | **4th location** |
| --- | --- | --- | --- | --- |
| Numbness/Loss of Sensation | hand (n>413) | foot/feet (n>336) | arm (n>336) | leg (n>201) |
| Coldness | foot/feet (n>356) | hand (n>237) | body (n>157) | arm (n>62) |
| Tingling/Prickling/Pins & Needles | hand (n>482) | foot/feet (n>432) | arm (n>355) | leg (n>252) |
| Electric Zap/Shock Sensation | leg (n>131) | arm (n>120) | foot/feet (n>93) | head (n>85) |
| Facial Paralysis | side (n>51) | cheek (n>26) | mouth (n>14) | eye/jaw* (n>13) |
| Facial Pressure/Numbness | sinus (n>32) | face (n>31) | side (n>29) | lips/head** (n>24) |
| Weakness | leg (n>377) | arm (n>319) | body (n>218) | hand (n>101) |

* Eye and jaw were reported equally for facial paralysis

** Lips and head were reported equally for facial pressure and numbness

*A.4 Systemic symptoms*Fatigue (98.3%, 95% confidence interval 97.9% to 98.7%) and post-exertional malaise (PEM) 89.0% (88.0% to 90.0%) were the most common symptoms reported by respondents (Figure 2a, Supplemental Table S8), as reported previously [[3]](https://www.zotero.org/google-docs/?M3riNZ). Each increased in likelihood over the first two months of illness before plateauing (Figure 4a). Weakness was experienced by 44.5% (42.9% to 46.1%) of respondents.

#

# Elevated temperature below 100.4F (58.2%, 56.5% to 59.8%) was almost twice as common as fever above 100.4F (30.8%, 29.3% to 32.3%). 3.0% (2.5% to 3.7%) experienced a continuous fever (>100.4F) for 3 or more months, and 15.0% (13.8% to 16.1%) experienced an elevated temperature, continuously, for 3 or more months.

#

# Skin sensations of burning, itching, or tingling without a rash were reported by 47.8% (45.3% to 48.5%) of respondents.

#

## *A.5 Reproductive/Genitourinary/Endocrine symptoms* Total of 2979 respondents reported that the question “If applicable, do you have periods/a menstrual cycle” applied to them by responding either *Yes*, *No - Post-Menopausal*, or *No - Other*. Of the 1792 respondents who reported having periods/a menstrual cycle, 36.1% (95% confidence interval 33.8% to 38.3%) reported experiencing menstrual/period issues. For this group, these issues included abnormally irregular periods (26.1%, 24.0% to 28.2%, Figure 2b, Supplemental Table S7) and abnormally heavy periods/clotting (19.7%, 18.0% to 21.6%). Of the 1123 cis women respondents over 49, 4.5% (3.46% to 5.85%) experienced post-menopausal bleeding/spotting. Of the 938 cis women respondents in their 40s, 3.0% (2.0% to 4.3%) experienced early menopause.

#

# Sexual dysfunction occurred across genders, experienced by 14.6% (95% confidence interval 12.1% to 17.4%) of men (cis or trans) respondents, 7.9% (7.0% to 9.0%) of women (cis or trans) respondents, and 15.87% (7.94% to 26.9%) out of 63 nonbinary respondents.

#

# Pain in testicles was reported by 10.9% (8.6% to 13.2%) of the 714 cis men participants.

#

# Extreme thirst was reported by 35.8% (34.3% to 37.3%) of respondents. Bladder control issues were experienced by 14.1% (13.1% to 15.3%) of respondents. Bladder control issues were not particularly variable over time (Figure 4b), while menstrual issues ramped up in prevalence over the first two months.

#

## *A.6 Cardiovascular symptoms* 86% (95% confidence interval 84.9% to 87.2%) of respondents reported experiencing cardiovascular symptoms (Figure 2c, Supplemental Table S8). The most commonly reported symptoms were heart palpitations (67.4%, 65.9% to 68.8%), tachycardia (61.4%, 59.8% to 62.9%), and pain/burning in the chest (53.1%, 51.5% to 54.7%). Fainting was experienced by 12.9% (11.9% to 14%) of respondents.

#

# Cardiovascular symptoms were more common over the first 2 months than in later months (Figure 4c). Even so, 40.1% (37.9% to 44.1%) of respondents with symptoms for over 6 months experienced heart palpitations, 33.7% (30.8% to 36.8%) experienced tachycardia, and 23.7% (20.7% to 26.0%) experienced pain/burning in the chest in month 7.

#

## *A.7 Musculoskeletal symptoms* Musculoskeletal symptoms were common in this cohort, seen in 93.9% (95% confidence 93.0% to 94.6%) (Figure 2d, Supplemental Table S9). Chest tightness was most common (74.8%, 73.4% to 76.1%), followed by muscle aches (69.1%, 67.6% to 70.6%) and joint pain (52.2%, 50.5% to 53.8%). In month 7, chest tightness affected 32.9% (29.9% to 36.0%) of month 7 respondents and muscle aches affected 43.7% (40.6% to 46.9%) of month 7 respondents (Figure 4d).

#

#

## *A.8 Immunologic and Autoimmune symptoms* Immunologic and autoimmune symptoms were reported by 21.0% (95% confidence interval 19.8% to 22.4%) of respondents (Figure 2e, Supplemental Table S10). Heightened reaction to old allergies was most common, at 12.1% (11.0% to 13.1%), followed by new allergies at 9.3% (8.4% to 10.2%). New or unexpected anaphylaxis reactions were notable at 4.1% (3.5% to 4.7%). Change in prevalence over time was not notable (Figure 4e).

#

# 20.3% of respondents (n=765) reported experiencing changes in sensitivity to medications.

#

# *A.9 HEENT (Head, ears, eyes, nose, throat) symptoms*

# 28 symptoms were defined as symptoms of the head, ears, eyes, nose, and throat (HEENT) (Figure 2f, Supplemental Table S11). All respondents experienced at least one HEENT symptom.

#

# Sore throat was the most prevalent symptom (59.5%, 95% confidence interval 57.9% to 61.1%) which was reported almost twice as often as the next most prevalent symptom, blurred vision (35.7%, 34.2% to 37.3%). Within this category, symptoms involving vision were as common as other organs. Notably, 1.0% (0.7% to 1.4%) of participants reported total loss of vision (no data on the extension and duration of vision loss were collected).

#

# Ear and hearing issues (including hearing loss), other eye issues, and tinnitus became more common over the duration studied (Figure 4f). Tinnitus, for example, increased from 11.5% (10.5% to 12.5%) of all respondents reporting it in week 1 to 26.2% (23.5% to 29.1%) of respondents with symptoms for over 6 months reporting it in month 7.

#

## *A.10 Pulmonary and Respiratory symptoms* 93.0% (95% confidence interval 92.2% to 93.8%) of respondents reported pulmonary and respiratory symptoms (Figure 2g, Supplemental Table S12). Shortness of breath at 77.4% (76.1% to 78.8%) was more common than dry cough at 66.2% (64.7% to 67.7%) or breathing difficulty with normal oxygen levels at 60.4% (58.8% to 61.9%). Rattling of breath was reported by 17.0% (15.8% to 18.3%) of respondents.

#

# Dry cough was reported by half of respondents in week 1 (50.6%, 49.0% to 52.5%) and week 2 (50.0%, 48.4% to 51.6%), and decreased to 20.1% (17.8% to 22.8%) of respondents with symptoms for over 6 months in month 7 (Figure 4g). Shortness of breath and breathing difficulties with normal oxygen increased from week 1 to week 2 and had relatively slow decline after month 2. Shortness of breath remained prevalent in 37.9% of respondents (34.8% to 41.0%) with symptoms in month 7 (Figure 11a).

#

## *A.11 Gastrointestinal symptoms* Gastrointestinal symptoms (Figure 2h, Supplemental Table S13) were reported at 85.5% (95% confidence interval 84.4% to 86.6%) overall. Diarrhea was the most commonly reported gastrointestinal symptom, experienced by 59.7% (58.1% to 61.3%) of respondents, followed by loss of appetite (51.6%, 50.0% to 53.2%) and nausea (47.8%, 46.2% to 49.4%). Of respondents experiencing symptoms after month 6, 20.5% (18.1% to 23.2%) reported diarrhea and 13.7% (11.6% to 16.0%) reported loss of appetite in month 7 as shown in Figure 4h.

#

#

## *A.12 Dermatologic symptoms* As shown in Figure 2i (Supplemental Table S14), dermatological symptoms were present in 59.1% (95% confidence interval 57.5% to 60.6%) of respondents. Itchy skin (31.2%, 29.7% to 32.6%) and skin rashes (27.8%, 26.3% to 29.2%) were most common. 17.8% (16.6% to 19.1%) of respondents reported petechiae, while COVID toe was reported by 13.0% (12.0% to 14.1%) of respondents. COVID toe, petechiae, and skin rashes were most likely to be reported in months 2 through 4 and decreased thereafter (Figure 4i).

## *A.13 Neuropsychiatric symptoms (others)* **Headaches** Headaches were reported by 77.0% of participants (95% confidence interval 75.4% to 78.0%, Supplemental Table S20), with the most common manifestations being ocular 40.9% (38.6 to 41.7%), diffuse 35.0% (33.6% to 36.7%), and temporal 34.0% (32.4% to 35.5%) (Figure 3f). 24.0% (22.5% to 25.2%) of respondents reported headaches after thinking/mental exertion and 23.0% (21.9% to 24.6%) experienced migraines. Of those experiencing migraines, 56.4% did not list migraines as a pre-existing condition. 46% of all respondents reported headaches during week 1, 54% of respondents experiencing symptoms in month 4 reported headaches in month 4, and 50% of respondents experiencing symptoms in month 7 reported headaches in month 7 (Figure 4l).

###

### **Emotion and mood** Changes to emotion and mood were reported by 88.3% (95% confidence interval 87.2% to 89.3%Supplemental Table S21) of participants (Figure 3a). Anxiety was the most common psychological symptom reported at 57.9% (56.4% to 59.5%), followed by irritability at 51.0% (49.5% to 52.7%). Depression was reported by 47.3% (45.7% to 48.9%) with 39.2% (37.6% to 40.7%) experiencing apathy. Mood lability, assessed by “mood swings” and “difficulty controlling emotions,” was reported by 46.3% (37.6% to 40.7%). Suicidality was reported by 11.6% (10.6% to 12.6%), and mania and hypomania were reported at 2.6% (2.1% to 3.1%) and 3.4% (2.8% to 4.0%), respectively. Of those who reported anxiety, 61.4% (59.4% to 63.4%) had no anxiety disorder prior to COVID. Of those who reported depression, 53.12% (50.8% to 55.4%) had no depressive disorder prior to COVID.

#

### **Taste and smell** Changes to taste and smell (Figure 3g) were reported by 57.6% (95% confidence interval 56.0% to 59.2%, Supplemental Table S22), with no significant difference seen in loss of smell (35.9%, 34.4% to 37.5%) vs. loss of taste (33.7%, 32.2% to 35.2%, p > 0.1, chi-squared test). Altered sense of taste was experienced by 25.1% (23.7% to 26.4%) of respondents, phantom smells (i.e. olfactory hallucinations or phantosmia) by 23.2% (21.9% to 24.6%) of respondents, and altered sense of smell by19.8% (18.5% to 21.1%) of respondents. Phantom smells were accompanied by a write-in question asking for a description of the smells, in which the most common words were “smoke,” “burning,” “cigarette,” and “meat.”

#

# Changes to smell and taste were more likely to occur earlier in the illness course, with 33.2% occuring in week 1. 25.2% (22.5% to 28%) of respondents with symptoms for over 6 months experienced changes to taste and smell in month 7 (Figure 4k).

### **Hallucinations** The most common hallucination reported was olfactory hallucinations 23.2% (21.9% to 24.6%, Supplemental Table S23), mentioned above (Figure 3i). Visual hallucinations were reported by 10.4% (9.5% to 11.4%) of respondents, auditory hallucinations by 6.5% (5.7% to 7.3%), and tactile hallucinations by 3.1% (2.6% to 3.7%).

#

#

#

# Appendix B: Supplementary Methods

*B.1 Symptom prevalence*

Excluded symptoms were high blood pressure, low blood pressure, thrombosis, confirmed seizures, (confirmed or suspected seizures), low oxygen levels, high blood sugar, and low blood sugar. The remaining 66 symptoms (out of 74) were included in analysis of the timeline of disease progression over 7 months (see below, Figure 4). Each symptom was further categorized into one of ten organ systems (Supplemental material, Appendix A), which were visualized as groups. The respondents for certain symptoms (non-primary language and reproductive/genitourinary symptoms) consisted of the subset of total respondents whom the symptom could apply to (i.e. those who spoke more than one language, cisgender female or non-binary and menstruating, cisgender female or non-binary and above or below 40 years of age, and male). Therefore, the symptom prevalence was calculated within the relevant subsample.

*B.2 Symptom prevalence over time*

Respondents indicated whether each of these symptoms was present during a series of time intervals following the onset of their first symptoms: week 1 (days 1-7), week 2 (days 8-14), week 3 (days 15-21), week 4 (days 22-30), month 2 (days 31-60), month 3 (days 61-90), month 4 (days 91-120), month 5 (days 121-150, month 6 (days 151-180), and month 7 (days 181-210).The time course of each symptom was defined as the probability of experiencing the symptom in each time interval, given that: 1) recovery had not occurred prior to the end of the interval, and 2) the symptom was applicable (menstruation-related symptoms are presented only for menstruating respondents). Probabilities were estimated for each interval as the fraction of respondents who experienced the symptom, among those who satisfied the two criteria above. The number of qualifying respondents in each time interval is given in Table S2.

*Table S2. Number of qualifying respondents in each time interval*

| **Symptoms**  **(66)** | **Week 1** | **Week 2** | **Week 3** | **Week 4** | **Month 2** | **Month 3** | **Month 4** | **Month 5** | **Month 6** | **Month 7** |
| --- | --- | --- | --- | --- | --- | --- | --- | --- | --- | --- |
| Menstruation Related (1) | 1792 | 1792 | 1792 | 1792 | 1757 | 1726 | 1704 | 1615 | 1190 | 462 |
| Other Symptoms (65) | 3762 | 3762 | 3762 | 3762 | 3681 | 3624 | 3563 | 3352 | 2454 | 966 |

*B.3 Symptom severity and count*

Overall symptom severity for each time interval (weeks 1-4, month 2-7) was measured using Likert scale (no symptom, very mild, mild, moderate, severe, very severe). The probability of each Likert option was calculated as the fraction of qualifying participants (as described above) who selected that option (Figure 1b). Total number of experienced symptoms (from the subset of 66) was measured for each qualifying respondent in each time interval. The mean value of symptom count was then calculated by averaging over all qualifying respondents.

*B.4 Statistical tests*
For Mann-Whitney U test, as well as Chi-squared test and Fisher’s exact test we used standard built-in MATLAB functions. To Bonferroni correct the Fisher’s exact tests, the output p-values were multiplied by the number of comparisons (for instance, number of symptoms).

*B.5 Stratification based on the diagnostic test time*

Test time, defined as the number of days between first experiencing symptoms and receiving a diagnostic test (RT-PCR or antigen), was significantly shorter in respondents with positive results compared to those with negative results (a median of 6 days for those who tested positive and 43 days for those who tested negative, p < 0.001, Mann-Whitney U test, see *Symptoms by test results,* and Supplemental Figure S6). To compare the symptom prevalence and time course (Supplemental Figure S7-S8), the two groups were stratified based on the test time by including respondents who were tested within certain time windows after illness onset. For prevalence estimates, time windows were ‘smaller than 10 days’, ‘between 10 and 20 days’, and ‘larger than 20 days’. For time course estimates, we limited the comparison to those who have been tested within 20 days after illness onset. Data stratification based on test time was done only for the diagnostic test.

*B.6 Text analysis*

The survey asked respondents to elaborate on their experience in free text for the following areas: body parts for sensorimotor symptoms, brain fog and memory issues, most debilitating symptoms, other diagnosis post illness, and work status. Deductive thematic analysis was used to tag and extract themes around impact on work [(27)](https://www.zotero.org/google-docs/?Kk2K7T). For textual input on participants’ experience of symptoms, such as cognitive dysfunction, a range of quotes was selected to provide a deepened understanding of the diversity of experiences [(25)](https://www.zotero.org/google-docs/?AJ5VVF). Identifying data were anonymized and longer sentences were truncated for brevity.

For the sensorimotor textual input questions, which asked which body part was affected, natural language processing was used in Python. The text was converted to lowercase, stripped of punctuation and extra whitespace, and stopwords were removed (using the original stopwords list from the Natural Language Toolkit ( NLTK) library as well as common non-symptom text inputs [(26)](https://www.zotero.org/google-docs/?bQQZek). The grammatical parts of speech were identified using a word tokenizer, and only nouns were reserved. The nouns were run through a translation function to convert all non-English nouns to their English counterparts, then counted, and the top four body parts were added to the table. The answers to “most debilitating symptoms'' followed a similar process, without the parts-of-speech tagging; another function was written to group similar descriptions (e.g. cognition, brain fog, and difficulty concentrating all went under “cognitive dysfunction”).

*B.6.1 Thematic analysis of “impact on work”*Respondents had the option to elaborate on their situation in free text, and these responses captured the precariousness of working with Long COVID (see selected quotes in Appendix D). From the thematic analysis of the participants’ free text responses (see Methods and selected quotes above), Several themes emerged through thematic analysis: At least 45% of working respondents were working remotely at the time of the survey, and it was noted how critical this was to respondents’ continued ability to work. Teleworking enabled respondents to take breaks when necessary and saved them the physical exertion of commuting to work. Respondents mentioned asking for other accommodations at work like flextime or moving to a role with lower physical or mental strain. Even with telecommuting, phased returns, and other accommodations, respondents commented on how difficult it was for them to work full or part-time, but described their financial need to do so.

It is important to note that the survey captured only a moment in time. Respondents described taking months of leave before going back to work either full-time or at reduced hours. Further, there were respondents who indicated that they tried to go back to work for several weeks but then relapsed or were unable to complete their work satisfactorily.

#

Appendix C: Participant profile

## *C.1 Socioeconomic status*

3084 (82.0%) participants reported their income at the time of the survey. A majority of participants in the USA, UK, and Canada belong to the middle and upper-middle income brackets, with 51.0% of participants in the USA earning more than $85,000/year and 22.5% earning more than $150,000/year. Meanwhile, 25.0% of survey participants from elsewhere reported less than €20,000/year in income and 51.1% reported earning less than €40,000/year (Figure S1).

## *C.2 Pre-existing conditions*

Most patients (83%) reported at least one pre-existing condition. The most commonly reported pre-existing conditions were seasonal allergies (36.3%), environmental allergies (24.1%), migraines (18.7%), and asthma (17.1%). Other conditions of note include acid reflux (12.2%), irritable bowel syndrome (12.9%), vitamin D deficiency (11.8%), obesity (10.7%), hypertension (9.1%), hyperlipidemia (7.4%), and myalgic encephalomyelitis / chronic fatigue syndrome (2.5%). In the United States, the prevalence of asthma is 7.7%. While this cohort is not representative of the U.S. population, the prevalence of asthma (17.07%) should be noted.

*Table S3. Pre-existing conditions reported by respondents*

| **Pre-existing Condition** | **Number of Respondents** | **Percentage** |
| --- | --- | --- |
| **High Risk*** | | |
| Cancer | 100 | 2.7% |
| Chronic Kidney Disease | 14 | 0.4% |
| COPD | 29 | 0.8% |
| Obesity | 401 | 10.8% |
| Auto-Immune / Rheumatologic Conditions | 257 | 6.9% |
| Type 2 Diabetes | 51 | 1.4% |
| Anemia | 190 | 5.10% |
| **Increased Risk*** | | |
| Asthma | 642 | 17.2% |
| Hypertension / High Blood Pressure | 344 | 9.2% |
| Dementia | 1 | 0.0% |
| Peripheral Neuropathy | 80 | 2.2% |
| Type 1 Diabetes | 13 | 0.4% |
| **Allergies** | | |
| Food | 604 | 16.2% |
| Environmental | 906 | 24.3% |
| Chemical | 231 | 6.2% |
| Seasonal | 1365 | 36.6% |
| Unknown Origin | 131 | 3.5% |
| Other Allergies not listed | 287 | 7.7% |
| **Other Pre-conditions** | | |
| Migraines | 702 | 18.6% |
| Vitamin D Deficiency | 442 | 11.9% |
| High Cholesterol / Hyperlipidemia | 279 | 7.5% |
| Recurrent Bacterial or Viral Infections | 159 | 4.4% |
| Acid Reflux Disease | 460 | 12.4% |
| ME/CFS | 95 | 2.6% |

* Risk assessment based on Centers for Disease Control and Prevention [(51)](https://www.zotero.org/google-docs/?vkYNq6)

## *C.3 Post-acute diagnoses*

1146 respondents (30.5%) sought a diagnosis after the onset of illness. 802 respondents received one of the following diagnoses listed in the table below. Additionally, 197 respondents received a diagnosis of post-viral fatigue, post-viral syndrome, post-viral inflammation, post-COVID fatigue syndrome, or post-COVID syndrome, pointing to different working diagnoses for Long COVID [(5,10)](https://www.zotero.org/google-docs/?jOyfga).

In text input, some respondents documented difficulties in receiving a diagnosis, with reasons ranging from a lack of access to specialists, waiting for tests to be scheduled and performed, to healthcare providers dismissing symptoms as anxiety.

*Table S4. Diagnosis reported by respondents after the onset of illness*

| **Diagnosis** | **Number of Respondents**  **(N=802)** | **% of Respondents** |
| --- | --- | --- |
| Migraine | 219 | 27.3% |
| POTS | 155 | 19.3% |
| Costochondritis | 146 | 18.2% |
| ME/CFS* | 118 | 14.7% |
| Psychiatric Diagnosis | 101 | 12.6% |
| Myocarditis | 71 | 8.6% |
| Neuralgia | 66 | 8.2% |
| Blood clot | 63 | 7.7% |
| Asthma | 55 | 6.9% |
| Small fiber neuropathy | 42 | 5.4% |
| Anxiety | 42 | 5.2% |
| Autonomic neuropathy | 40 | 5.0% |
| Polyneuropathy | 31 | 3.9% |
| Pericarditis | 31 | 3.9% |
| Tachycardia | 25 | 3.1% |
| Stroke | 20 | 2.5% |
| Traumatic Brain Injury (TBI)** | 20 | 2.5% |
| GERD*** | 20 | 2.5% |
| Encephalitis | 18 | 2.2% |
| Pleurisy | 18 | 2.2% |

* Myalgic Encephalomyelitis / Chronic Fatigue Syndrome

** Including TBI-like symptoms

*** Gastroesophageal Reflux Disease

##

## *C.4 Survey distribution*

The majority of respondents came from the Body Politic COVID-19 Slack support group, Long COVID Support group on Facebook, and Long Haul Covid Fighters support group on Facebook. Additionally, the survey was shared with international advocacy groups, including #ApresJ20 (France), Long Covid SOS/Long Covid International, Apuakoronaan (Finland), COVID Persistente Espana, COVID-19 Persistent Madrid, Long COVID ACTS (Spain), and Long Covid Italia. Additional support groups included Pós-Covid-19 (Brazil), Covid Survivor Indonesia, Young Covid Survivors, Black Covid-19 Survivors, COVID-19 Vi som är drabbade (Sweden), BIPOC Women Covid Long Hauler Support Group, Survivor Corps, and others. It was also shared on other social media platforms, including Instagram, Twitter, and Reddit, and with nonprofits and mutual aid organizations. Additionally, it was shared with the Body Politic and Patient-Led Research Collaborative team email mailing groups reaching over 15,774 contacts.

*Table S5. Countries or Regions reported by respondents*

| **Country or Geographic Region** | **Number of Respondents** | **Countries or Geographic Regions with N<3 Respondents** |
| --- | --- | --- |
| United States of America | 1567 | Ecuador |
| United Kingdom | 1316 | India |
| France | 163 | Indonesia |
| Canada | 155 | Czech Republic |
| Spain | 99 | Hungary |
| Netherlands | 61 | Viet Nam |
| Ireland | 58 | Bangladesh |
| Sweden | 55 | San Marino |
| Russian Federation | 32 | Dominican Republic |
| Germany | 31 | Syrian Arab Republic |
| Belgium | 29 | Estonia |
| Australia | 23 | Cuba |
| Switzerland | 21 | Kenya |
| New Zealand | 16 | Senegal |
| Brazil | 16 | Ukraine |
| Norway | 14 | Serbia |
| Italy | 13 | Malta |
| Finland | 10 | South Korea |
| South Africa | 7 | Costa Rica |
| United Arab Emirates | 6 | Pakistan |
| Austria | 6 | Kazakhstan |
| Japan | 5 | Uzbekistan |
| Denmark | 4 | China |
| Greece | 4 | Slovenia |
| Slovakia | 3 | Colombia |
| Argentina | 3 |  |
| Israel | 3 |  |
| Egypt | 3 |  |
| Philippines | 3 |  |
| Portugal | 3 |  |
| Mexico | 3 |  |

# Appendix D: Quotes from Participants

| **Cognitive Dysfunction and Memory Loss** |
| --- |
| *“mother has started to help me take the medications I’m on because I* ***can’t remember*** *if I’ve taken them immediately after having the bottle in my hand”*  *“was trying to fill out a mortgage application form and couldn’t remember our rent. I put £3750 a month. My partner said, no it’s £1375. So I put £13750. My partner said no, so I tried several more times - I* ***was just guessing numbers****”*  *“sitting on the toilet to pee and* ***had to stop for a second to think*** *if I was really there and not about to pee myself or the bed”*  *"****don't remember what I did*** *in March or April up until the last week of April. I had almost nothing on my schedule. I don't know what I did”*  *“put food on the gas stove and walked away for over an hour,* ***only noticing when they were smoking/burning****”*  *"****forget how to do normal routines*** *like running a meeting at work"*  *“****felt lost driving*** *and had to stop and find my position in a GPS to be able to drive back home. It's a route I have done hundreds of times”*  *"have trouble* ***comprehending new ideas****”*  *"****can't hold multiple trains of thought*** *[...] If I tell myself I have to water my plants, I must do it before another thought comes into my mind because otherwise I will forget"*  *“****can't follow plots*** *in movies or tv shows,* ***have to write everything down****, have to remember to look at notes”*  *“had to terminate many phone calls because I* ***could no longer comprehend the speakers nor communicate clearly*** *with them”*  *“used to do the New York Times crossword puzzle every single day and I* ***can't even manage the mini ones*** *now”*  *"****can't focus on reading complex texts****, and it makes me feel very tired to do that"*  *“Found that I had become dyslexic - and knew it was happening at the time,* ***could not remember how to spell words*** *- also found I was missing words from sentences and sometimes writing things that did not make sense”* |
|  |
|  |
|  |
|  |

| **Impact on Work** |
| --- |
| *“I* ***worked at some point for a few weeks, in June, but had to stop*** *(couldn't handle a conversation on the phone without brain fog / feeling dizzy / heavy breath trouble because of talking) after a few minutes”*  *“Haven't been able to work for [...] months due to brain fog.* ***Was supposed to go back last week on reduced hours. I resigned instead.*** *I have worked there as Director of [...] for just over [...] years.”*  *“Still on medical leave. Unpaid and* ***denied short term disability****.”*  *“I* ***went from [being] a workaholic to no workaholic at all****. This is the extreme opposite of who I am. [...] I do not know the person I have become.”*  *“I* ***went back to work too soon and wish I hadn’t. Finally had to take a 5 week break*** *in July/ August with the support of my employer. This helped a lot. I have now been back at work for 5 weeks and my symptoms have got worse to a degree.”*  *“I had to take two weeks off, had to work from home for four, but had to return for two weeks with fever as* ***my employer would not give me more time*** *[...].”*  *“I* ***asked to reduce hours*** *or work more from home to which* ***it was denied****.”*  *“I've been working from home. Haven't officially reduced my hours, but* ***my boss [has] been flexible and encouraged me to rest when needed****.”*  *“While* ***I've been able to keep my job while working from home****, I must admit that if it were not so, I would most definitely NOT be able to work at all.* ***I can barely leave my bedroom on most days****.”*  *“I* ***have needed more-flexible hours*** *(working remotely) post-COVID. That way,* ***I can rest as needed throughout the day****. If I had to return to in-person work at this point, it would be severely reduced hours if at all.”* |
|  |
|  |
|  |
|  |

Appendix E: Supporting Figures

**Figure S1. Income brackets by country.** Stacked bar chart compares income brackets of participants from top countries and worldwide. Note that income brackets differ between a and b.

**Figure S2 – Figure 1 extension. a.** Survival function for Male vs Female**.** (Kaplan-Meier estimator), characterizing the distribution of disease duration for Female (blue) and Male (orange) respondents. b. Average symptom severity over time, for “Recovered” (green, ) and “Not recovered” participants. c. Average number of reported symptoms over time, for “recovered” (green) and “Not recovered” participants. Shaded regions represent 95% simultaneous confidence bands

**Figure S3 - normalized probability of symptoms over time.** Heatmap shows the normalized probability of each symptom from week 1 to month 7. Rows are sorted using multidimensional scaling, to capture similarity in time course shapes such that similar shapes are adjacent.

**

**Figure S4 - symptom break in 4.4% of respondents.** 164 out of 3762 subjects (4.4%) had a temporary break in symptoms, limited to the resolution available(the first 4 weeks, and one data point for each month, until month seven). Yellow shows "symptom present", dark blue indicates "temporary break" which is defined as a window between two "symptom present" time points. Light blue shows "symptom absent" i.e. a time point with no symptom, that has "symptom present" on only one side of it (either preceding or following). Light blue at the beginning indicates either that the symptoms started later, or respondents experienced symptoms not asked in the survey. Light blue at the end, indicates either recovery, or that the symptom went away at least until month 7. The right plot shows the probability of temporary breaks (dark blues), over all patients.

**

**Figure S5 - prevalence of symptoms removed from the main analyses.** Eight symptoms were excluded, as their measurement required specialized equipment or tests that many participants may not have had access to. Excluded symptoms included 1. high blood pressure, 2. low blood pressure, 3. thrombosis, 4. confirmed seizures, 5. suspected seizures, 6. low oxygen levels, 7. high blood sugar, and 8. low blood sugar. Error bars are bootstrap 95% confidence intervals.

**Figure S6 - Test time.** (a) Number of days between first experiencing symptoms and receiving a diagnostic test (RT-PCR or antigen) for Positively (blue) vs Negatively tested (grey) respondents. (b) similar to a, for antibody testing.


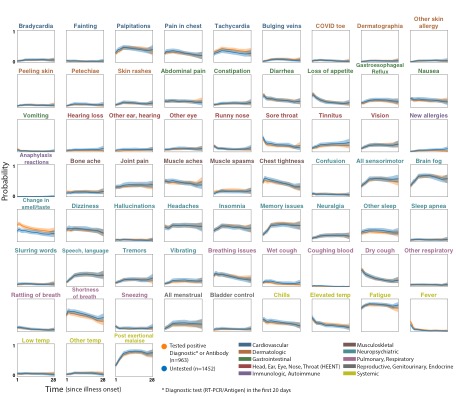


**Figure S7 - Symptom time courses for respondents with positive results vs. untested respondents.** Plots show symptom time courses (similar to Fig. 7) for respondents who were confirmed COVID-positive via diagnostic or antibody testing (orange) vs those without any diagnostic or antibody testing (blue). Shaded regions show simultaneous 95% confidence bands (over time and symptoms). Symptom names are colored according to the affected organ systems.


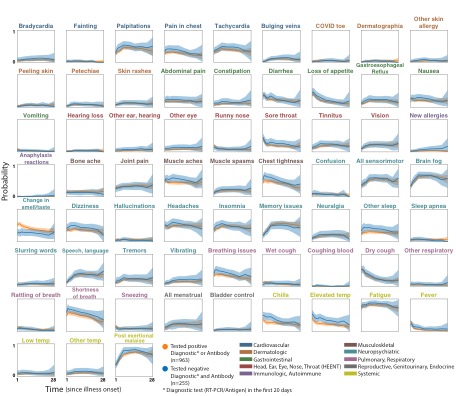


**Figure S8 - Symptom time courses for respondents with positive vs. negative test results.** Plots show symptom time courses (similar to Fig. 7) for respondents who were confirmed COVID-positive via diagnostic or antibody testing (orange) vs those with negative diagnostic and antibody test results (blue). Shaded regions show simultaneous 95% confidence bands (over time and symptoms). Symptom names are colored according to the affected organ systems.


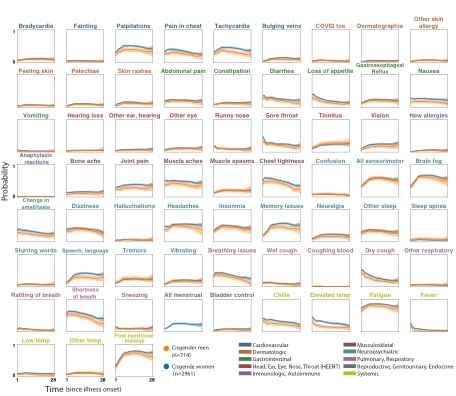


**Figure S9 - Symptom time courses for cisgender female vs. cisgender male respondents.** Plots show symptom time courses (similar to Fig. 7) for cisgender women (blue) and cisgende men (orange). Shaded regions show simultaneous 95% confidence bands (over time and symptoms). Symptom names are colored according to the affected organ systems.

#

#

*Appendix F: Raw Data Tables*

*Table S6. Systemic Symptom Prevalence Data*

| Symptom | Total # | Mean Prevalence | Lower CI | Upper CI |
| --- | --- | --- | --- | --- |
| Chills/flushing/sweats | 2124 | 56.46 | 54.86 | 58.03 |
| Coldness | 1261 | 33.52 | 32.06 | 35.03 |
| Elevated temperature (98.8-100.4 F) | 2188 | 58.16 | 56.54 | 59.75 |
| Fatigue | 3699 | 98.33 | 97.87 | 98.70 |
| Fever (>100.4 F) | 1158 | 30.78 | 29.32 | 32.27 |
| Heat intolerance | 1024 | 27.22 | 25.84 | 28.65 |
| Low temperature | 776 | 20.63 | 19.30 | 21.96 |
| Night sweats | 1535 | 40.80 | 39.21 | 42.40 |
| Other temperature problems | 586 | 15.60 | 14.51 | 16.83 |
| Post-Exertional Malaise | 3350 | 89.05 | 88.04 | 90.03 |
| Skin sensations: burning, tingling, or itchiness without rash | 1766 | 46.94 | 45.32 | 48.54 |
| Temperature lability | 1539 | 40.91 | 39.34 | 42.45 |
| Weakness | 1675 | 44.52 | 42.93 | 46.07 |

*Table S7. Reproductive/Genitourinary/Endocrine Symptom Prevalence Data*

| Symptom | Number of Respondents | Relevant Respondents | Mean Prevalence | Lower CI | Upper CI |
| --- | --- | --- | --- | --- | --- |
| Abnormally heavy periods/clotting | 355 | 1792** | 19.81 | 17.98 | 21.63 |
| Abnormally irregular periods | 474 | 1792** | 26.45 | 24.03 | 28.20 |
| All menstrual/period issues - with menstrual cycles | 632 | 1792** | 36.07 | 33.79 | 38.23 |
| All menstrual/period issues - post-menopausal and no/other menstrual cycles | 78 | 1970^~^ | 4.54 | 3.47 | 5.88 |
| All Reproductive | 1746 | 3762 | 46.41 | 44.82 | 47.98 |
| Decrease in size of testicles/penis cis_men | 23 | 714^ | 3.22 | 2.09 | 4.76 |
| Decrease in size of testicles/penis nonbinary | 1 | 63*^ | 1.59 | 0.00 | 8.04 |
| Early Menopause cis_women_in_40s | 28 | 938^+^ | 2.99 | 2.02 | 4.25 |
| Early Menopause cis_women_under_40 | 3 | 900*** | 0.33 | 0.11 | 0.89 |
| Extreme thirst | 1346 | 3762 | 35.78 | 34.26 | 37.29 |
| High blood sugar (if measured) | 164 | 3762 | 4.36 | 3.75 | 5.05 |
| Low blood sugar (if measured) | 65 | 3762 | 1.73 | 1.33 | 2.18 |
| Other menstrual issues | 303 | 1609* | 18.83 | 16.01 | 20.43 |
| Other semen/penis/testicles issues cis_men | 35 | 714^ | 4.90 | 3.48 | 6.69 |
| Other semen/penis/testicles issues nonbinary | 1 | 63*^ | 1.59 | 0.00 | 7.94 |
| Pain in testicles cis_men | 78 | 714^ | 10.92 | 8.64 | 13.23 |
| Pain in testicles nonbinary | 2 | 63*^ | 3.17 | 0.00 | 11.11 |
| Post-Menopausal bleeding/spotting cis_women_in_40s | 12 | 938^+^ | 1.28 | 0.64 | 2.23 |
| Post-Menopausal bleeding/spotting cis_women_over_49 | 51 | 1123^++^ | 4.54 | 3.46 | 5.86 |
| Reproductive and Urinary Symptoms - Bladder control issues | 532 | 3762 | 14.14 | 13.05 | 15.26 |
| Sexual dysfunction - cis men | 104 | 714^ | 14.57 | 12.04 | 17.23 |
| Sexual dysfunction - trans men | 1 | 4^+++^ | 0.25 | 0 | 75 |
| Sexual dysfunction - cis women | 236 | 2961^~~^ | 7.97 | 7.06 | 8.98 |
| Sexual dysfunction - trans women | 1 | 8^^ | 12.50 | 0 | 50 |
| Sexual dysfunction - nonbinary | 10 | 63*^ | 15.87 | 7.94 | 26.98 |
| Urinary issues, other | 565 | 3762 | 15.02 | 13.93 | 16.19 |
| Decrease in size of testicles/penis trans_women | 0 | 8^^ |  |  |  |
| Early Menopause nonbinary | 0 | 63*^ |  |  |  |
| Early Menopause trans_men | 0 | 4^+++^ |  |  |  |
| Other semen/penis/testicles issues trans_women | 0 | 8^^ |  |  |  |
| Pain in testicles trans_women | 0 | 8^^ |  |  |  |
| Post-Menopausal bleeding/spotting nonbinary | 0 | 63*^ |  |  |  |
| Post-Menopausal bleeding/spotting trans_men | 0 | 4^~^ |  |  |  |

* total respondents to whom the “have menstrual cycle” question applied

** total respondents who had a menstrual cycle

*** total cis women respondents under age 40

^+^ total cis women respondents ages 40-49

^++^ total cis women respondents over age 49

^+++^ total trans men respondents

^~^ total post-menopausal or other menstrual cycle respondents

^~~^ total cis women respondents

*^ total nonbinary respondents

^ total cis men respondents

^^ total trans women respondents

*Table S8. Cardiovascular Symptom Prevalence Data*

| Symptom | Number of Respondents | Mean Prevalence | Lower CI | Upper CI |
| --- | --- | --- | --- | --- |
| Abnormally high blood pressure | 755 | 20.07 | 18.79 | 21.35 |
| Abnormally low blood pressure | 443 | 11.78 | 10.77 | 12.84 |
| Blood clots (Thrombosis) | 131 | 3.48 | 2.95 | 4.12 |
| Bradycardia (low heart rate, <60 beats per minute) | 658 | 17.49 | 16.27 | 18.69 |
| Fainting | 486 | 12.92 | 11.88 | 14.01 |
| Heart palpitations | 2534 | 67.36 | 65.87 | 68.82 |
| Pain/burning in chest | 1997 | 53.08 | 51.54 | 54.68 |
| Tachycardia | 2308 | 61.35 | 59.78 | 62.89 |

*Table S9. Musculoskeletal Symptom Prevalence Data*

| Symptom | Number of Respondents | Mean Prevalence | Lower CI | Upper CI |
| --- | --- | --- | --- | --- |
| Joint pain | 1962 | 52.15 | 50.53 | 53.77 |
| Muscle aches | 2601 | 69.14 | 67.62 | 70.60 |
| Bone ache or burning | 910 | 24.19 | 22.83 | 25.57 |
| Muscle spasms | 1222 | 32.48 | 30.97 | 33.97 |
| Stiff neck | 1471 | 39.10 | 37.59 | 40.70 |
| Tightness of Chest | 2813 | 74.77 | 73.37 | 76.13 |

*Table S10. Immunologic/Autoimmune Symptom Prevalence Data*

| Symptom | Number of Respondents | Mean Prevalence | Lower CI | Upper CI |
| --- | --- | --- | --- | --- |
| Changes in sensitivity to medication | 765 | 20.33 | 19.06 | 21.64 |
| Dermatographia (writing on your skin causes red lines where you scratched) | 288 | 7.66 | 6.83 | 8.53 |
| Heightened reaction to old allergies | 454 | 12.07 | 11.03 | 13.13 |
| New allergies (food, chemical, environmental, etc) | 348 | 9.25 | 8.37 | 10.18 |
| New/unexpected anaphylaxis reaction | 153 | 4.07 | 3.48 | 4.73 |
| Shingles | 104 | 2.76 | 2.26 | 3.32 |

*Table S11. HEENT (Head, Ear, Eyes, Nose, Throat) Symptom Prevalence Data*

| Symptom | Number of Respondents | Mean Prevalence | Lower CI | Upper CI |
| --- | --- | --- | --- | --- |
| Bloodshot eyes | 579 | 15.39 | 14.25 | 16.56 |
| Changes in the voice | 1012 | 26.9 | 25.49 | 28.31 |
| Changes to the ear canal (such as pressure, blockage, burning, swelling) | 822 | 21.85 | 20.55 | 23.18 |
| Dry eyes | 1077 | 28.63 | 27.19 | 30.12 |
| Ear and Hearing Symptoms - Hearing loss | 326 | 8.67 | 7.79 | 9.60 |
| Ear and Hearing Symptoms - Other ear/hearing issues | 467 | 12.41 | 11.27 | 13.40 |
| Ear pain | 971 | 25.81 | 24.43 | 27.22 |
| Eye pressure or pain | 992 | 26.37 | 24.99 | 27.78 |
| Facial paralysis (please indicate where on face was paralyzed) | 127 | 3.38 | 2.84 | 3.99 |
| Floaters | 755 | 20.07 | 18.77 | 21.35 |
| Itchy eyes | 909 | 24.16 | 22.78 | 25.52 |
| Lump in throat/difficulty swallowing | 1222 | 32.48 | 30.99 | 34.00 |
| Numbness/loss of sensation in/near the ear | 176 | 4.68 | 4.04 | 5.37 |
| Other eye issues | 465 | 12.36 | 11.35 | 13.45 |
| Pink eye (conjunctivitis) | 560 | 14.89 | 13.77 | 16.08 |
| Redness on the outside of eyes | 317 | 8.43 | 7.57 | 9.33 |
| Runny nose | 1102 | 29.29 | 27.90 | 30.78 |
| Seeing things in your peripheral vision | 502 | 13.34 | 12.31 | 14.46 |
| Sensation of facial pressure/numbness, left side | 309 | 8.21 | 7.36 | 9.12 |
| Sensation of facial pressure/numbness, other: | 228 | 6.06 | 5.34 | 6.86 |
| Sensation of facial pressure/numbness, right side | 254 | 6.75 | 5.98 | 7.58 |
| Sensitivity to light | 1159 | 30.81 | 29.35 | 32.32 |
| Sneezing | 989 | 26.29 | 24.91 | 27.70 |
| Sore Throat | 2239 | 59.52 | 57.93 | 61.08 |
| Blurred vision | 1343 | 35.7 | 34.18 | 37.27 |
| Double vision | 260 | 6.91 | 6.11 | 7.76 |
| Total loss of vision | 37 | 0.98 | 0.69 | 1.36 |
| Tunnel vision | 125 | 3.32 | 2.79 | 3.91 |
| Vision symptoms, overall | 1269 | 33.73 | 32.27 | 35.27 |

*Table S12. Pulmonary Symptom Prevalence Data*

| Symptom | Number of Respondents | Mean Prevalence | Lower CI | Upper CI |
| --- | --- | --- | --- | --- |
| Cough with mucus production | 1062 | 28.23 | 26.82 | 29.72 |
| Dry cough | 2491 | 66.21 | 64.67 | 67.70 |
| Episodes of breathing difficulty/gasping for air when your oxygen saturation is normal | 2271 | 60.37 | 58.80 | 61.94 |
| Low oxygen levels (<94%)* | 996 | 26.48 | 25.07 | 27.91 |
| Coughing up Blood | 194 | 5.16 | 4.49 | 5.90 |
| Other Respiratory and Sinus Symptoms | 359 | 9.54 | 8.64 | 10.50 |
| Rattling of breath | 641 | 17.04 | 15.84 | 18.26 |
| Shortness of Breath | 2913 | 77.43 | 76.08 | 78.76 |

* Measurement required specialized equipment or tests that many participants may not have had access to.

*Table S13. Gastrointestinal Symptom Prevalence Data*

| Symptom | Number of Respondents | Mean Prevalence | Lower CI | Upper CI |
| --- | --- | --- | --- | --- |
| Abdominal pain | 1492 | 39.66 | 38.12 | 41.23 |
| Constipation | 930 | 24.72 | 23.34 | 26.10 |
| Diarrhea | 2246 | 59.7 | 58.13 | 61.27 |
| Feeling full quickly when eating | 1158 | 30.78 | 29.32 | 32.22 |
| Hyperactive bowel sensations | 881 | 24.00 | 22.62 | 25.33 |
| Loss of Appetite | 1942 | 51.62 | 49.95 | 53.22 |
| Lower Esophagus Burning / gastroesophageal reflux / acid reflux | 1317 | 35.01 | 33.47 | 36.52 |
| Nausea | 1797 | 47.77 | 46.17 | 49.36 |
| Vomiting | 539 | 14.33 | 13.24 | 15.47 |

*Table S14. Dermatologic Symptom Prevalence Data*

| Symptom | Number of Respondents | Mean Prevalence | Lower CI | Upper CI |
| --- | --- | --- | --- | --- |
| Brittle/discolored nail | 358 | 9.52 | 8.59 | 10.45 |
| Itchy skin | 1172 | 31.15 | 29.72 | 32.64 |
| Itchy, other | 192 | 5.1 | 4.44 | 5.85 |
| COVID toes (discoloration, swelling, painful, or blistering toes) | 490 | 13.02 | 11.99 | 14.14 |
| Other Skin and Allergy symptoms | 383 | 10.18 | 9.25 | 11.18 |
| Peeling skin | 488 | 12.97 | 11.91 | 14.06 |
| Petechiae (tiny purple, red, or brown spots on the skin, usually on arms, legs, stomach, buttocks, and occasionally inside mouth or on eyelids) | 671 | 17.84 | 16.64 | 19.09 |
| Skin rashes | 1045 | 27.78 | 26.34 | 29.24 |
| Visibly inflamed/bulging veins | 726 | 19.3 | 18.08 | 20.57 |

*Table S15. Neuropsychiatric-Cognitive Functioning Symptom Prevalence Data*

| Symptom | Number of Respondents | Mean Prevalence | Lower CI | Upper CI |
| --- | --- | --- | --- | --- |
| Acute (sudden) confusion/disorientation | 691 | 18.37 | 17.15 | 19.64 |
| Agnosia (failure to recognize or identify objects despite intact sensory functioning) | 345 | 9.17 | 8.27 | 10.13 |
| Cognitive Dysfunction, overall (Brain fog) | 3203 | 85.14 | 83.94 | 86.26 |
| Difficulty problem-solving or decision-making | 2034 | 54.07 | 52.42 | 55.61 |
| Difficulty thinking | 2444 | 64.97 | 63.40 | 66.43 |
| Difficulty with executive functioning (planning, organizing, figuring out the sequence of actions, abstracting) | 2166 | 57.58 | 56.01 | 59.14 |
| Other Cognitive Functioning Symptoms | 323 | 8.59 | 7.71 | 9.52 |
| Poor attention or concentration | 2814 | 74.8 | 73.42 | 76.21 |
| Slowed thoughts | 1572 | 41.79 | 40.22 | 43.43 |
| Thoughts moving too quickly | 570 | 15.15 | 14.04 | 16.35 |

*Table S16. Neuropsychiatric-Memory Symptom Prevalence Data*

| Symptom | Number of Respondents | Mean Prevalence | Lower CI | Upper CI |
| --- | --- | --- | --- | --- |
| Forgetting how to do routine tasks (tying your shoe laces, washing your hands) | 453 | 12.04 | 11.00 | 13.08 |
| Long-term memory loss (long-term memory can be anything from remembering yesterday, forgetting you’ve done a task, forgetting recently learned information, or forgetting your third-grade experience) | 1359 | 36.12 | 34.64 | 37.64 |
| Inability to make new memories | 275 | 7.31 | 6.49 | 8.19 |
| Other Memory Symptoms | 507 | 13.47 | 12.44 | 14.59 |
| Short-term memory loss (memory that lasts ~30 seconds, i.e. remembering a phone number before writing it down, or forgetting you’re in the middle of a task) | 2438 | 64.81 | 63.34 | 66.37 |

*Table S17. Neuropsychiatric-Speech and Language Symptom Prevalence Data*

| Symptom | Number of Respondents | Mean Prevalence | Lower CI | Upper CI |
| --- | --- | --- | --- | --- |
| Changes to non-primary (second/third) language skills* | 191 | 28.85 | 27.12 | 184 |
| Difficulting speaking in complete sentences | 835 | 22.2 | 20.87 | 23.52 |
| Difficulty communicating in writing | 615 | 16.35 | 15.18 | 17.54 |
| Difficulty communicating verbally | 1099 | 29.21 | 27.78 | 30.68 |
| Difficulty finding the right words while speaking/writing | 1743 | 46.33 | 44.79 | 47.93 |
| Difficulty processing/understanding what others say | 894 | 23.76 | 22.46 | 25.17 |
| Difficulty reading/processing written text | 931 | 24.75 | 23.34 | 26.08 |
| Other Speech/Language Symptoms | 233 | 6.19 | 5.45 | 6.99 |
| Slurring words/speech | 594 | 15.79 | 14.62 | 16.99 |
| Speaking unrecognizable words | 334 | 8.88 | 8.00 | 9.81 |

*Estimated probability for multilingual participants (n=662)

*Table S18. Neuropsychiatric-Sensorimotor Symptom Prevalence Data*

| Symptom | Number of Respondents | Mean Prevalence | Lower CI | Upper CI |
| --- | --- | --- | --- | --- |
| Dizziness / vertigo / unsteadiness or balance issues | 2531 | 67.28 | 65.76 | 68.77 |
| Electrical zaps/electrical shock sensation | 945 | 25.12 | 23.76 | 26.53 |
| Inability to cry | 184 | 4.89 | 4.25 | 5.61 |
| Inability to yawn | 250 | 6.65 | 5.87 | 7.47 |
| Neuralgia (nerve pain) | 1177 | 31.29 | 29.82 | 32.80 |
| Numbness/loss of sensation | 1332 | 35.41 | 33.86 | 36.90 |
| Numbness/weakness on one side of the body only | 472 | 12.55 | 11.48 | 13.61 |
| Seizures (confirmed) | 22 | 0.58 | 0.37 | 0.88 |
| Seizures (suspected) | 102 | 2.71 | 2.23 | 3.27 |
| Sensation of brain pressure | 1227 | 32.62 | 31.15 | 34.21 |
| Sensation of brain warmth/"on fire" | 461 | 12.25 | 11.22 | 13.32 |
| Sensitivity to noise | 1305 | 34.69 | 33.15 | 36.20 |
| Tingling/prickling/pins and needles sensation | 1852 | 49.23 | 47.66 | 50.82 |
| Tinnitus | 1280 | 34.02 | 32.56 | 35.57 |
| Tremors | 1511 | 40.16 | 38.62 | 41.76 |
| Vibrating sensations | 1610 | 42.80 | 40.34 | 44.13 |

*Table S19. Neuropsychiatric-Sleep Symptom Prevalence Data*

| Symptom | Number of Respondents | Mean Prevalence | Lower CI | Upper CI |
| --- | --- | --- | --- | --- |
| Awakened by feeling like you couldn’t breathe | 1355 | 36.02 | 34.53 | 37.59 |
| Difficulty falling asleep | 1489 | 39.58 | 37.99 | 41.12 |
| Insomnia | 2582 | 68.63 | 67.12 | 70.12 |
| Lucid dreams (dreams where you are aware you are dreaming or have some control over what you dream about) | 579 | 15.39 | 14.22 | 16.56 |
| Nightmares | 966 | 25.68 | 24.30 | 27.14 |
| Other Sleeping Issues Symptoms | 608 | 16.16 | 14.99 | 17.38 |
| Restless leg syndrome | 668 | 17.76 | 16.56 | 19.03 |
| Sleep apnea | 267 | 7.1 | 9.46 | 11.46 |
| Vivid dreams | 1239 | 32.93 | 31.45 | 34.45 |
| Waking up early in the morning | 936 | 24.88 | 23.50 | 26.26 |
| Waking up several times during the night | 1791 | 47.61 | 46.01 | 49.18 |

*Table S20. Neuropsychiatric-Headaches Symptom Prevalence Data*

| Symptom | Number of Respondents | Mean Prevalence | Lower CI | Upper CI |
| --- | --- | --- | --- | --- |
| Headaches, at the base of the skull | 1012 | 26.9 | 25.52 | 28.31 |
| Headaches, behind the eyes | 1509 | 40.11 | 38.57 | 41.68 |
| Headaches, diffuse (entire brain) | 1322 | 35.14 | 33.57 | 36.68 |
| Headaches, in the temples | 1276 | 33.92 | 32.43 | 35.46 |
| Headaches, other | 488 | 12.97 | 11.94 | 14.06 |
| Headaches/pain after mental exertion | 897 | 23.84 | 22.49 | 25.23 |
| Migraines | 872 | 23.18 | 21.85 | 24.56 |

*Table S21. Neuropsychiatric-Emotion and Mood Symptom Prevalence Data*

| Symptom | Number of Respondents | Mean Prevalence | Lower CI | Upper CI |
| --- | --- | --- | --- | --- |
| Aggression | 280 | 7.44 | 6.65 | 8.32 |
| Anger | 911 | 24.22 | 22.86 | 25.62 |
| Anxiety | 2179 | 57.92 | 56.35 | 59.52 |
| Apathy (lack of feeling, emotion, interest, or concern) | 1473 | 39.15 | 37.56 | 40.72 |
| Delusions | 112 | 2.98 | 2.47 | 3.56 |
| Depression | 1779 | 47.29 | 45.72 | 48.88 |
| Euphoria (a feeling or state of intense excitement and happiness) | 188 | 5 | 4.33 | 5.72 |
| Feeling like the world isn’t real (derealization) or Feeling like you aren’t real/like you’re observing yourself from outside your body (depersonalization) | 1000 | 26.58 | 25.20 | 28.07 |
| Hypomania (a milder form of mania) | 128 | 3.4 | 2.84 | 4.01 |
| Impulsivity and Disinhibition | 451 | 11.99 | 10.95 | 13.05 |
| Irritability | 1924 | 51.14 | 49.52 | 52.71 |
| Mania (abnormally elevated/excited mood, decreased need for sleep, occasionally with delusions) | 96 | 2.55 | 2.07 | 3.08 |
| Mood Lability | 1743 | 46.33 | 44.71 | 47.90 |
| Other Emotional Changes Symptoms | 231 | 6.14 | 5.40 | 6.94 |
| Sense of doom | 1269 | 33.73 | 32.22 | 35.25 |
| Suicidality | 436 | 11.59 | 10.61 | 12.63 |
| Tearfulness | 1599 | 42.5 | 40.91 | 44.10 |

*Table S22. Neuropsychiatric-Taste and Smell Symptom Prevalence Data*

| Symptom | Number of Respondents | Mean Prevalence | Lower CI | Upper CI |
| --- | --- | --- | --- | --- |
| Altered sense of smell | 745 | 19.8 | 18.53 | 21.08 |
| Altered sense of taste | 943 | 25.07 | 23.68 | 26.45 |
| Heightened sense of smell | 323 | 8.59 | 7.71 | 9.49 |
| Heightened sense of taste | 101 | 2.68 | 2.18 | 3.22 |
| Loss of smell | 1352 | 35.94 | 34.42 | 37.51 |
| Loss of taste | 1267 | 33.68 | 32.16 | 35.22 |
| Phantom smells (imagining/hallucinating smells - smelling things that aren't there) | 872 | 23.18 | 21.88 | 24.56 |
| Phantom taste (imagining/hallucinating tastes - tasting things when there's nothing in your mouth) | 339 | 9.01 | 8.16 | 9.97 |

*Table S23. Neuropsychiatric-Hallucinations Symptom Prevalence Data*

| Symptom | Number of Respondents | Mean Prevalence | Lower CI | Upper CI |
| --- | --- | --- | --- | --- |
| Auditory (hearing) Hallucinations | 244 | 6.49 | 5.74 | 7.31 |
| Hallucinations, other | 87 | 2.31 | 1.86 | 2.84 |
| Tactile (touch) Hallucinations | 116 | 3.08 | 2.55 | 3.69 |
| Visual (seeing) Hallucinations | 391 | 10.39 | 9.46 | 11.43 |

Table S24. Symptom Prevalence Timecourse Data: Mean (Lower CI, Upper CI)

| Symptom | W1 | W2 | W3 | W4 | M2 | M3 | M4 | M5 | M6 | M7 |
| --- | --- | --- | --- | --- | --- | --- | --- | --- | --- | --- |
| Abdominal pain | 18.66 (17.44, 19.94) | 19.40 (18.18, 20.68) | 19.83 (18.61, 21.13) | 20.87 (19.59, 22.17) | 22.47 (21.15, 23.86) | 21.14 (19.87, 22.51) | 21.13 (19.84, 22.49) | 20.05 (18.75, 21.42) | 18.13 (16.67, 19.71) | 19.15 (16.77, 21.66) |
| Abnormally high blood pressure* | 6.35 (5.61, 7.18) | 7.20 (6.41, 8.05) | 8.00 (7.15, 8.90) | 8.56 (7.68, 9.46) | 11.17 (10.20, 12.23) | 10.35 (9.41, 11.36) | 9.68 (8.75, 10.65) | 8.68 (7.76, 9.65) | 8.76 (7.69, 9.92) | 7.87 (6.28, 9.67) |
| Abnormally low blood pressure* | 3.32 (2.79, 3.93) | 4.09 (3.51, 4.76) | 4.63 (3.99, 5.32) | 4.92 (4.25, 5.66) | 5.60 (4.87, 6.37) | 6.37 (5.63, 7.19) | 6.43 (5.66, 7.27) | 6.32 (5.55, 7.20) | 6.07 (5.19, 7.07) | 5.59 (4.25, 7.18) |
| Acute (sudden) confusion/disorientation | 7.31 (6.51, 8.21) | 8.83 (7.95, 9.78) | 8.69 (7.81, 9.65) | 8.32 (7.44, 9.25) | 8.99 (8.08, 9.94) | 8.39 (7.51, 9.32) | 8.17 (7.31, 9.13) | 7.40 (6.54, 8.32) | 7.58 (6.59, 8.67) | 4.97 (3.76, 6.53) |
| All menstrual/period issues - with menstrual cycles** | 9.30 (8.02, 10.78) | 10.73 (9.34, 12.25) | 12.44 (10.94, 14.02) | 15.53 (13.88, 17.19) | 24.45 (22.51, 26.51) | 25.37 (23.37, 27.44) | 25.27 (23.23, 27.34) | 23.16 (21.15, 25.25) | 21.34 (19.06, 23.71) | 22.05 (18.41, 26.07) |
| Select sensorimotor symptoms*** | 31.95 (30.49, 33.44) | 36.39 (34.85, 37.93) | 40.80 (39.23, 42.34) | 44.13 (42.53, 45.69) | 55.15 (53.57, 56.77) | 58.86 (57.28, 60.52) | 59.11 (57.49, 60.68) | 57.13 (55.44, 58.74) | 53.22 (51.17, 55.15) | 52.07 (48.82, 55.29) |
| Bladder control issues | 4.31 (3.69, 5.00) | 5.24 (4.57, 5.98) | 5.72 (5.00, 6.49) | 6.33 (5.58, 7.15) | 8.07 (7.23, 9.00) | 8.20 (7.33, 9.15) | 8.53 (7.66, 9.51) | 8.23 (7.33, 9.18) | 7.78 (6.75, 8.86) | 8.49 (6.78, 10.32) |
| Blood clots (Thrombosis)* | 0.51 (0.32, 0.77) | 0.56 (0.35, 0.82) | 0.90 (0.64, 1.25) | 1.04 (0.74, 1.38) | 1.49 (1.12, 1.93) | 1.57 (1.19, 2.02) | 1.18 (0.87, 1.59) | 0.92 (0.63, 1.30) | 0.98 (0.65, 1.44) | 1.66 (0.96, 2.60) |
| Bone ache or burning | 11.96 (10.93, 13.00) | 13.00 (11.94, 14.09) | 13.05 (12.01, 14.17) | 13.40 (12.33, 14.51) | 14.75 (13.63, 15.95) | 15.01 (13.87, 16.20) | 14.99 (13.82, 16.18) | 14.62 (13.47, 15.86) | 13.81 (12.52, 15.21) | 17.29 (15.00, 19.80) |
| Bradycardia | 5.13 (4.44, 5.85) | 5.79 (5.08, 6.57) | 6.17 (5.42, 6.97) | 6.99 (6.22, 7.81) | 9.18 (8.27, 10.13) | 9.99 (9.03, 10.98) | 10.86 (9.87, 11.90) | 11.31 (10.25, 12.41) | 9.74 (8.61, 10.97) | 8.18 (6.57, 10.05) |
| Brain fog | 31.23 (29.74, 32.72) | 36.71 (35.17, 38.22) | 43.51 (41.95, 45.08) | 48.03 (46.41, 49.63) | 63.71 (62.20, 65.30) | 66.67 (65.13, 68.19) | 66.35 (64.77, 67.89) | 62.86 (61.20, 64.53) | 56.85 (54.90, 58.78) | 55.49 (52.45, 58.75) |
| Breathing difficulty (normal O2 saturation level) | 34.18 (32.64, 35.70) | 42.05 (40.40, 43.59) | 42.80 (41.17, 44.36) | 41.17 (39.63, 42.72) | 42.38 (40.81, 43.96) | 37.64 (36.08, 39.21) | 33.20 (31.67, 34.76) | 28.52 (27.05, 30.08) | 27.10 (25.37, 28.89) | 21.33 (18.90, 24.01) |
| Changes to sense of smell and taste | 33.23 (31.74, 34.72) | 37.61 (36.07, 39.15) | 36.90 (35.35, 38.44) | 34.72 (33.23, 36.26) | 34.09 (32.57, 35.64) | 30.74 (29.28, 32.29) | 28.40 (26.95, 29.94) | 25.45 (24.02, 27.01) | 23.43 (21.77, 25.17) | 25.16 (22.45, 27.98) |
| Chills/flushing/sweats | 39.93 (38.41, 41.47) | 39.21 (37.69, 40.72) | 36.23 (34.72, 37.77) | 34.48 (32.99, 35.99) | 35.97 (34.42, 37.50) | 32.06 (30.57, 33.58) | 29.83 (28.37, 31.36) | 28.04 (26.51, 29.59) | 26.00 (24.29, 27.74) | 20.19 (17.70, 22.73) |
| Constipation | 7.42 (6.62, 8.29) | 8.40 (7.55, 9.33) | 9.38 (8.51, 10.34) | 9.81 (8.90, 10.82) | 12.88 (11.85, 14.04) | 13.52 (12.43, 14.68) | 13.25 (12.18, 14.41) | 13.04 (11.90, 14.17) | 13.00 (11.72, 14.37) | 13.25 (11.23, 15.59) |
| Cough with mucus production | 10.66 (9.71, 11.70) | 12.57 (11.54, 13.66) | 13.34 (12.28, 14.43) | 13.42 (12.36, 14.57) | 13.91 (12.81, 15.06) | 10.79 (9.79, 11.83) | 10.02 (9.07, 11.03) | 9.37 (8.42, 10.39) | 8.15 (7.15, 9.29) | 10.35 (8.54, 12.38) |
| Coughing up Blood | 1.09 (0.80, 1.46) | 1.67 (1.30, 2.10) | 1.65 (1.28, 2.10) | 1.70 (1.33, 2.15) | 1.82 (1.42, 2.28) | 1.19 (0.88, 1.58) | 0.95 (0.67, 1.31) | 0.84 (0.57, 1.19) | 0.69 (0.41, 1.08) | 1.14 (0.61, 1.98) |
| COVID toe | 3.11 (2.58, 3.69) | 3.93 (3.32, 4.60) | 5.05 (4.39, 5.79) | 5.45 (4.78, 6.25) | 6.79 (6.02, 7.64) | 5.93 (5.18, 6.74) | 5.05 (4.36, 5.81) | 4.00 (3.36, 4.68) | 3.26 (2.62, 4.03) | 3.21 (2.23, 4.47) |
| Dermatographia | 2.23 (1.78, 2.74) | 2.58 (2.10, 3.11) | 2.98 (2.47, 3.56) | 3.38 (2.82, 3.99) | 4.78 (4.13, 5.50) | 5.19 (4.51, 5.96) | 4.94 (4.28, 5.70) | 4.98 (4.30, 5.78) | 4.24 (3.50, 5.11) | 4.97 (3.70, 6.47) |
| Diarrhea | 34.05 (32.59, 35.59) | 34.53 (33.04, 36.07) | 29.53 (28.07, 31.05) | 27.94 (26.53, 29.45) | 29.80 (28.36, 31.29) | 25.19 (23.79, 26.63) | 23.74 (22.38, 25.19) | 21.48 (20.12, 22.91) | 19.52 (18.03, 21.10) | 20.50 (18.06, 23.18) |
| Dizziness / vertigo / unsteadiness or balance issues | 35.73 (34.18, 37.27) | 39.21 (37.72, 40.83) | 39.90 (38.36, 41.52) | 39.77 (38.22, 41.36) | 42.81 (41.22, 44.43) | 41.78 (40.16, 43.37) | 39.97 (38.36, 41.58) | 37.80 (36.16, 39.41) | 35.25 (33.36, 37.16) | 27.02 (24.28, 29.81) |
| Dry cough | 50.58 (48.99, 52.18) | 50.05 (48.43, 51.63) | 45.22 (43.65, 46.81) | 39.98 (38.44, 41.57) | 34.94 (33.41, 36.49) | 26.93 (25.53, 28.41) | 23.58 (22.23, 25.04) | 20.38 (19.06, 21.80) | 18.01 (16.53, 19.56) | 20.19 (17.68, 22.78) |
| Elevated temperature (98.8-100.4F) | 43.09 (41.49, 44.68) | 39.15 (37.56, 40.70) | 33.81 (32.30, 35.35) | 30.49 (29.03, 32.00) | 30.62 (29.13, 32.13) | 25.94 (24.51, 27.44) | 24.11 (22.71, 25.52) | 22.52 (21.14, 23.93) | 21.27 (19.68, 22.92) | 17.18 (14.90, 19.67) |
| Fainting | 3.75 (3.16, 4.39) | 3.80 (3.22, 4.44) | 3.85 (3.27, 4.52) | 3.85 (3.27, 4.52) | 4.86 (4.18, 5.59) | 4.61 (3.94, 5.31) | 3.96 (3.34, 4.63) | 3.82 (3.19, 4.50) | 3.18 (2.52, 3.93) | 3.42 (2.37, 4.72) |
| Fatigue | 69.11 (67.62, 70.55) | 74.69 (73.31, 76.05) | 77.22 (75.86, 78.52) | 79.59 (78.28, 80.86) | 84.49 (83.29, 85.63) | 85.02 (83.82, 86.15) | 84.54 (83.32, 85.71) | 82.76 (81.46, 84.01) | 77.91 (76.26, 79.52) | 77.74 (74.98, 80.31) |
| Fever (>= 100.4F) | 23.84 (22.49, 25.22) | 19.30 (18.08, 20.60) | 12.20 (11.16, 13.24) | 9.01 (8.11, 9.94) | 8.01 (7.19, 8.96) | 5.55 (4.84, 6.34) | 4.41 (3.79, 5.12) | 4.24 (3.60, 4.95) | 3.91 (3.19, 4.76) | 2.90 (1.96, 4.12) |
| Hallucinations | 4.65 (4.01, 5.37) | 5.58 (4.89, 6.35) | 5.77 (5.05, 6.54) | 5.40 (4.70, 6.14) | 7.14 (6.36, 8.00) | 6.84 (6.06, 7.69) | 6.29 (5.55, 7.12) | 5.82 (5.07, 6.67) | 5.62 (4.80, 6.62) | 6.21 (4.82, 7.88) |
| Headaches and related symptoms | 45.77 (44.26, 47.40) | 45.22 (43.65, 46.81) | 44.66 (43.06, 46.23) | 44.98 (43.41, 46.54) | 53.00 (51.45, 54.64) | 53.31 (51.66, 54.90) | 53.66 (52.04, 55.29) | 52.57 (50.91, 54.23) | 50.61 (48.64, 52.61) | 49.59 (46.48, 52.80) |
| Hearing loss | 2.98 (2.47, 3.54) | 3.27 (2.74, 3.85) | 3.80 (3.22, 4.44) | 3.88 (3.30, 4.55) | 4.86 (4.19, 5.60) | 5.10 (4.44, 5.89) | 5.22 (4.53, 6.00) | 5.16 (4.45, 5.96) | 5.30 (4.41, 6.19) | 6.42 (5.00, 8.07) |
| Heart palpitations | 28.44 (26.98, 29.88) | 32.80 (31.31, 34.32) | 37.80 (36.23, 39.34) | 40.27 (38.68, 41.84) | 48.55 (46.94, 50.16) | 48.73 (47.12, 50.39) | 47.77 (46.13, 49.42) | 45.14 (43.43, 46.84) | 40.99 (39.06, 42.97) | 40.89 (37.85, 44.05) |
| High blood sugar (if measured)* | 1.28 (0.96, 1.67) | 1.46 (1.12, 1.89) | 1.49 (1.12, 1.91) | 1.57 (1.20, 2.02) | 1.85 (1.44, 2.31) | 1.90 (1.49, 2.38) | 1.80 (1.39, 2.27) | 1.73 (1.32, 2.21) | 1.75 (1.26, 2.32) | 1.24 (0.65, 2.12) |
| Insomnia | 34.08 (32.54, 35.57) | 37.59 (36.02, 39.07) | 41.55 (39.98, 43.09) | 43.43 (41.81, 44.98) | 50.75 (49.15, 52.33) | 50.03 (48.41, 51.64) | 48.41 (46.80, 50.06) | 46.00 (44.30, 47.65) | 42.54 (40.59, 44.47) | 42.55 (39.51, 45.65) |
| Joint pain | 26.95 (25.52, 28.41) | 28.73 (27.33, 30.20) | 29.45 (28.02, 30.97) | 30.09 (28.68, 31.61) | 35.15 (33.69, 36.74) | 35.98 (34.46, 37.56) | 37.36 (35.74, 38.91) | 36.46 (34.85, 38.11) | 34.43 (32.59, 36.40) | 37.06 (34.15, 40.15) |
| Loss of Appetite | 36.07 (34.56, 37.59) | 38.04 (36.50, 39.61) | 33.84 (32.35, 35.41) | 30.20 (28.73, 31.71) | 25.54 (24.15, 26.99) | 19.76 (18.47, 21.10) | 17.49 (16.23, 18.74) | 15.18 (14.00, 16.42) | 13.57 (12.25, 14.92) | 13.66 (11.60, 15.95) |
| Low blood sugar (if measured)* | 0.35 (0.19, 0.58) | 0.37 (0.21, 0.61) | 0.56 (0.35, 0.85) | 0.53 (0.35, 0.82) | 0.79 (0.54, 1.11) | 0.83 (0.58, 1.18) | 0.73 (0.48, 1.04) | 0.54 (0.33, 0.83) | 0.49 (0.25, 0.82) | 0.31 (0.10, 0.87) |
| Low oxygen levels (O2 saturation<94%)* | 11.86 (10.85, 12.92) | 15.23 (14.09, 16.42) | 15.44 (14.30, 16.61) | 14.09 (13.00, 15.20) | 14.48 (13.39, 15.64) | 11.62 (10.61, 12.71) | 9.91 (8.96, 10.92) | 8.44 (7.53, 9.41) | 7.58 (6.57, 8.67) | 5.90 (4.58, 7.55) |
| Low temperature | 9.09 (8.21, 10.07) | 9.62 (8.75, 10.63) | 9.60 (8.69, 10.58) | 9.81 (8.90, 10.85) | 11.41 (10.40, 12.46) | 10.46 (9.49, 11.46) | 9.37 (8.46, 10.36) | 8.62 (7.68, 9.59) | 8.72 (7.66, 9.90) | 7.87 (6.30, 9.68) |
| Lower Esophagus Burning / refluxes | 12.41 (11.38, 13.50) | 13.90 (12.81, 15.02) | 15.68 (14.51, 16.85) | 17.60 (16.35, 18.82) | 21.95 (20.60, 23.27) | 23.01 (21.67, 24.39) | 22.51 (21.13, 23.90) | 22.34 (20.96, 23.71) | 20.90 (19.29, 22.55) | 19.98 (17.54, 22.63) |
| Memory issues | 19.70 (18.42, 20.95) | 23.15 (21.82, 24.48) | 28.44 (27.06, 29.93) | 33.39 (31.92, 34.90) | 50.29 (48.66, 51.94) | 54.33 (52.67, 55.97) | 55.91 (54.27, 57.50) | 53.46 (51.76, 55.16) | 49.39 (47.36, 51.35) | 50.52 (47.33, 53.64) |
| Muscle aches | 46.54 (44.98, 48.14) | 48.56 (47.00, 50.21) | 47.58 (46.01, 49.23) | 47.18 (45.61, 48.83) | 49.93 (48.37, 51.61) | 47.88 (46.27, 49.52) | 46.37 (44.75, 48.00) | 44.75 (43.09, 46.46) | 40.71 (38.78, 42.69) | 43.69 (40.64, 46.87) |
| Muscle spasms | 12.25 (11.24, 13.32) | 13.40 (12.36, 14.51) | 15.18 (14.09, 16.40) | 16.29 (15.15, 17.52) | 20.02 (18.76, 21.34) | 21.00 (19.74, 22.36) | 20.71 (19.39, 22.05) | 20.67 (19.33, 22.05) | 19.15 (17.62, 20.75) | 22.05 (19.44, 24.74) |
| Nausea | 24.83 (23.47, 26.24) | 25.39 (24.03, 26.79) | 24.08 (22.75, 25.49) | 23.29 (21.96, 24.67) | 25.54 (24.15, 27.02) | 24.31 (22.94, 25.73) | 23.29 (21.93, 24.73) | 22.73 (21.35, 24.21) | 21.43 (19.86, 23.09) | 19.88 (17.41, 22.42) |
| Neuralgia (nerve pain) | 10.10 (9.17, 11.11) | 11.83 (10.82, 12.92) | 13.37 (12.33, 14.49) | 15.20 (14.09, 16.40) | 20.02 (18.77, 21.32) | 22.49 (21.16, 23.86) | 22.76 (21.41, 24.16) | 22.23 (20.87, 23.65) | 21.27 (19.70, 22.93) | 17.49 (15.13, 19.98) |
| New allergies | 1.44 (1.09, 1.86) | 1.75 (1.38, 2.21) | 2.02 (1.62, 2.50) | 2.66 (2.18, 3.19) | 4.75 (4.09, 5.46) | 5.55 (4.85, 6.33) | 6.43 (5.67, 7.28) | 6.56 (5.75, 7.44) | 5.18 (4.35, 6.09) | 5.90 (4.50, 7.49) |
| New/unexpected anaphylaxis reaction | 0.43 (0.24, 0.66) | 0.64 (0.43, 0.93) | 0.88 (0.61, 1.22) | 1.17 (0.85, 1.54) | 1.71 (1.33, 2.18) | 1.60 (1.24, 2.06) | 1.91 (1.49, 2.40) | 1.79 (1.38, 2.29) | 1.92 (1.43, 2.53) | 1.55 (0.89, 2.44) |
| Other ear/hearing issues | 3.96 (3.38, 4.63) | 4.70 (4.07, 5.42) | 5.32 (4.63, 6.03) | 5.37 (4.68, 6.09) | 7.04 (6.24, 7.89) | 7.59 (6.76, 8.50) | 7.97 (7.12, 8.91) | 8.08 (7.19, 9.05) | 7.86 (6.85, 9.02) | 9.21 (7.52, 11.22) |
| Other eye symptoms | 7.95 (7.12, 8.85) | 8.29 (7.47, 9.22) | 8.64 (7.76, 9.54) | 9.12 (8.24, 10.05) | 10.98 (9.99, 12.01) | 12.25 (11.23, 13.35) | 12.04 (11.01, 13.12) | 11.72 (10.68, 12.83) | 11.33 (10.12, 12.63) | 13.25 (11.23, 15.49) |
| Other Respiratory and Sinus | 5.45 (4.76, 6.22) | 5.98 (5.24, 6.78) | 6.17 (5.42, 6.96) | 6.25 (5.50, 7.04) | 6.79 (6.01, 7.64) | 6.54 (5.79, 7.41) | 6.09 (5.36, 6.95) | 5.79 (5.04, 6.62) | 5.05 (4.26, 5.98) | 5.59 (4.29, 7.20) |
| Other Skin and Allergy | 2.60 (2.13, 3.14) | 3.03 (2.53, 3.62) | 3.69 (3.14, 4.36) | 4.33 (3.72, 5.02) | 6.06 (5.33, 6.88) | 6.51 (5.76, 7.40) | 6.54 (5.77, 7.40) | 6.59 (5.77, 7.47) | 4.93 (4.13, 5.86) | 4.87 (3.62, 6.34) |
| Other sleeping symptoms | 28.39 (26.95, 29.85) | 31.50 (30.02, 33.01) | 33.63 (32.11, 35.11) | 34.40 (32.88, 35.89) | 37.33 (35.75, 38.89) | 36.18 (34.57, 37.76) | 35.00 (33.43, 36.59) | 32.49 (30.92, 34.09) | 30.36 (28.57, 32.27) | 30.33 (27.43, 33.30) |
| Other temperature problems | 7.44 (6.65, 8.29) | 7.39 (6.59, 8.27) | 8.16 (7.34, 9.06) | 8.51 (7.66, 9.44) | 11.17 (10.19, 12.22) | 11.84 (10.84, 12.93) | 11.96 (10.93, 13.07) | 11.75 (10.71, 12.88) | 10.23 (9.09, 11.51) | 7.56 (6.04, 9.40) |
| Pain/burning in chest | 28.65 (27.22, 30.12) | 34.56 (33.04, 36.12) | 36.36 (34.85, 37.91) | 37.24 (35.70, 38.81) | 39.01 (37.48, 40.63) | 35.76 (34.20, 37.35) | 32.47 (30.95, 34.05) | 28.70 (27.22, 30.24) | 24.45 (22.79, 26.21) | 23.29 (20.74, 26.01) |
| Peeling skin | 3.11 (2.60, 3.69) | 3.77 (3.19, 4.41) | 4.97 (4.31, 5.69) | 5.26 (4.57, 6.01) | 6.90 (6.09, 7.74) | 6.73 (5.92, 7.57) | 6.29 (5.52, 7.12) | 5.91 (5.16, 6.76) | 5.05 (4.23, 5.98) | 5.59 (4.27, 7.17) |
| Petechiae | 3.83 (3.24, 4.49) | 4.44 (3.80, 5.13) | 5.72 (5.00, 6.49) | 7.10 (6.30, 7.95) | 9.89 (8.95, 10.88) | 10.13 (9.18, 11.15) | 10.08 (9.15, 11.09) | 9.16 (8.21, 10.15) | 6.81 (5.86, 7.88) | 7.76 (6.20, 9.59) |
| Post Exertional Malaise | 32.08 (30.62, 33.63) | 37.27 (35.75, 38.83) | 45.77 (44.18, 47.34) | 53.67 (52.10, 55.32) | 70.47 (68.98, 71.95) | 76.41 (75.01, 77.79) | 78.28 (76.90, 79.61) | 76.67 (75.23, 78.09) | 71.19 (69.43, 72.97) | 72.26 (69.35, 75.03) |
| Rattling of breath | 9.04 (8.16, 9.99) | 10.66 (9.70, 11.67) | 11.19 (10.23, 12.23) | 10.61 (9.65, 11.62) | 9.24 (8.33, 10.23) | 7.20 (6.40, 8.09) | 5.84 (5.11, 6.65) | 4.83 (4.15, 5.61) | 4.40 (3.64, 5.24) | 5.59 (4.23, 7.13) |
| Runny nose | 19.17 (17.89, 20.41) | 16.69 (15.50, 17.94) | 13.32 (12.25, 14.43) | 11.24 (10.29, 12.31) | 11.14 (10.15, 12.20) | 9.44 (8.53, 10.44) | 9.09 (8.19, 10.10) | 9.22 (8.28, 10.26) | 8.96 (7.88, 10.16) | 11.59 (9.71, 13.78) |
| Seizures (confirmed)* | 0.19 (0.08, 0.37) | 0.21 (0.11, 0.40) | 0.19 (0.08, 0.37) | 0.19 (0.08, 0.37) | 0.30 (0.16, 0.52) | 0.25 (0.11, 0.47) | 0.25 (0.11, 0.48) | 0.24 (0.12, 0.45) | 0.24 (0.08, 0.52) | 0.00 (0.00, 0.00) |
| Seizures (suspected)* | 0.48 (0.29, 0.74) | 0.40 (0.24, 0.64) | 0.53 (0.35, 0.80) | 0.43 (0.24, 0.66) | 0.81 (0.57, 1.14) | 1.05 (0.75, 1.42) | 1.15 (0.84, 1.54) | 0.95 (0.66, 1.32) | 0.90 (0.57, 1.32) | 1.04 (0.51, 1.85) |
| Shortness of Breath | 50.58 (48.94, 52.21) | 60.77 (59.17, 62.25) | 62.12 (60.58, 63.69) | 61.22 (59.65, 62.76) | 61.18 (59.59, 62.74) | 56.24 (54.57, 57.82) | 52.46 (50.80, 54.07) | 46.39 (44.66, 48.07) | 37.94 (35.99, 39.84) | 37.89 (34.87, 41.03) |
| Skin rashes | 6.94 (6.17, 7.76) | 8.21 (7.36, 9.12) | 9.84 (8.90, 10.79) | 11.00 (10.02, 12.04) | 15.24 (14.06, 16.39) | 15.04 (13.86, 16.23) | 14.45 (13.33, 15.63) | 12.11 (11.05, 13.26) | 10.64 (9.47, 11.91) | 12.32 (10.36, 14.52) |
| Sleep apnea | 4.81 (4.17, 5.53) | 5.53 (4.84, 6.30) | 6.03 (5.32, 6.86) | 6.46 (5.74, 7.31) | 7.53 (6.72, 8.43) | 7.01 (6.22, 7.88) | 6.37 (5.62, 7.24) | 6.15 (5.37, 7.01) | 6.19 (5.29, 7.21) | 6.94 (5.46, 8.67) |
| Slurring words/speech | 5.34 (4.65, 6.09) | 6.57 (5.82, 7.39) | 7.15 (6.35, 8.03) | 7.44 (6.65, 8.35) | 8.75 (7.86, 9.71) | 8.47 (7.61, 9.41) | 7.97 (7.11, 8.88) | 7.28 (6.42, 8.16) | 7.25 (6.27, 8.32) | 7.87 (6.28, 9.70) |
| Sneezing | 15.39 (14.25, 16.56) | 13.29 (12.25, 14.41) | 10.87 (9.89, 11.91) | 9.54 (8.61, 10.50) | 9.86 (8.92, 10.85) | 9.19 (8.26, 10.18) | 9.91 (8.92, 10.92) | 10.95 (9.92, 12.05) | 9.49 (8.37, 10.67) | 13.35 (11.36, 15.62) |
| Sore Throat | 45.48 (43.89, 47.08) | 37.56 (35.99, 39.10) | 30.33 (28.87, 31.84) | 27.22 (25.81, 28.65) | 27.71 (26.26, 29.12) | 24.12 (22.71, 25.52) | 23.97 (22.58, 25.39) | 22.58 (21.20, 24.03) | 18.42 (16.91, 20.01) | 20.60 (18.19, 23.26) |
| Speech/language issues | 13.02 (11.99, 14.14) | 15.63 (14.49, 16.83) | 21.24 (19.91, 22.54) | 25.15 (23.76, 26.56) | 36.29 (34.73, 37.89) | 39.62 (38.03, 41.26) | 40.08 (38.49, 41.73) | 38.22 (36.61, 39.92) | 35.17 (33.28, 37.08) | 37.99 (34.99, 41.03) |
| Tachycardia | 28.73 (27.32, 30.20) | 32.32 (30.86, 33.86) | 34.72 (33.23, 36.26) | 37.35 (35.83, 38.89) | 44.23 (42.65, 45.84) | 42.91 (41.28, 44.54) | 41.65 (40.03, 43.30) | 38.63 (36.98, 40.30) | 34.56 (32.70, 36.44) | 33.75 (30.80, 36.76) |
| Tightness of Chest | 46.41 (44.84, 48.01) | 55.95 (54.36, 57.52) | 57.52 (55.90, 59.12) | 56.09 (54.49, 57.68) | 56.21 (54.59, 57.78) | 51.57 (49.93, 53.19) | 47.71 (46.09, 49.34) | 42.45 (40.73, 44.11) | 33.54 (31.67, 35.42) | 32.92 (29.93, 35.97) |
| Tinnitus | 11.46 (10.47, 12.52) | 12.84 (11.80, 13.93) | 14.49 (13.40, 15.63) | 15.76 (14.62, 16.96) | 21.41 (20.12, 22.79) | 23.32 (22.01, 24.76) | 24.95 (23.57, 26.45) | 25.27 (23.78, 26.75) | 25.31 (23.53, 27.05) | 26.19 (23.51, 29.10) |
| Tremors | 11.78 (10.77, 12.81) | 13.10 (12.07, 14.22) | 14.94 (13.80, 16.08) | 16.96 (15.82, 18.18) | 23.28 (21.93, 24.67) | 24.25 (22.87, 25.68) | 25.15 (23.77, 26.62) | 23.81 (22.41, 25.28) | 22.49 (20.90, 24.18) | 25.16 (22.54, 28.03) |
| Vibrating Sensations | 8.75 (7.87, 9.70) | 10.37 (9.44, 11.38) | 11.88 (10.90, 12.97) | 13.34 (12.28, 14.46) | 18.64 (17.40, 19.92) | 19.81 (18.58, 21.14) | 20.85 (19.56, 22.22) | 20.05 (18.73, 21.40) | 18.66 (17.15, 20.24) | 19.46 (17.01, 22.03) |
| Visibly inflamed/bulging veins | 3.16 (2.63, 3.75) | 3.80 (3.22, 4.47) | 5.16 (4.49, 5.91) | 6.86 (6.09, 7.71) | 10.27 (9.29, 11.28) | 12.31 (11.25, 13.39) | 13.19 (12.10, 14.34) | 12.44 (11.33, 13.58) | 10.23 (9.07, 11.45) | 10.14 (8.41, 12.21) |
| Vision symptoms | 9.97 (9.04, 10.93) | 11.48 (10.47, 12.52) | 14.25 (13.18, 15.42) | 15.98 (14.83, 17.17) | 20.95 (19.67, 22.29) | 22.68 (21.36, 24.06) | 23.77 (22.41, 25.18) | 23.15 (21.74, 24.59) | 22.94 (21.34, 24.63) | 26.50 (23.76, 29.30) |
| Vomiting | 6.46 (5.72, 7.31) | 5.45 (4.73, 6.19) | 3.96 (3.38, 4.65) | 3.72 (3.14, 4.36) | 3.80 (3.21, 4.45) | 2.87 (2.37, 3.46) | 2.92 (2.41, 3.51) | 2.66 (2.16, 3.24) | 2.85 (2.25, 3.59) | 2.90 (1.97, 4.12) |

* Measurement required specialized equipment or tests that many participants may not have had access to

** Measured out of individuals with menstrual cycles, per table 2.

***This category includes weakness, numbness, tingling (or pins and needles, or other skin sensations), coldness, electrical zaps, facial pressure, facial paralysis
